# Radiofrequency cascade readout of coupled spin qubits

**DOI:** 10.1038/s41928-026-01582-8

**Published:** 2026-03-30

**Authors:** Jacob F. Chittock-Wood, Ross C. C. Leon, Michael A. Fogarty, Tara Murphy, Felix-Ekkehard von Horstig, Sofia M. Patomäki, Giovanni A. Oakes, James Williams, Nathan Johnson, Julien Jussot, Stefan Kubicek, Bogdan Govoreanu, David F. Wise, John J. L. Morton, M. Fernando Gonzalez-Zalba

**Affiliations:** 1 Quantum Motion, London, UK; 2 London Centre for Nanotechnology, University College London, London, UK; 3 Department of Materials Sciences and Metallurgy, University of Cambridge, Cambridge, UK; 4 IMEC, Leuven, Belgium; 5 Present Address: Center for Emergent Matter Science, RIKEN, Saitama, Japan; 6 Present Address: CIC nanoGUNE Consolider, Donostia-San Sebastian, Gipuzkoa, Spain; 7 Present Address: IKERBASQUE, Basque Foundation for Science, Bilbao, Spain

**Keywords:** Electrical and electronic engineering, Qubits, Quantum dots, Quantum information, Sensors

## Abstract

Silicon spin qubits based on metal–oxide–semiconductor (MOS) technology are compatible with semiconductor manufacturing and offer a route to scalable quantum processing. However, spin readout typically relies on proximal charge sensors, which add architectural complexity and limit qubit connectivity. In situ dispersive readout techniques are more compact, which can alleviate these constrains, but exhibit limited sensitivity. Here we report a radiofrequency electron-cascade readout method that enhances the dispersive signal through alternating-current electron co-tunnelling. With this approach, we achieve an enhancement in signal-to-noise ratio of more than 35 dB, leading to a minimum integration time of 7.6 ± 0.2 µs. We demonstrate singlet-triplet readout of two-electron spins in a natural silicon planar MOS quantum dot array, and coherent spin control using the exchange interaction, which forms the basis for entangling gates. We find dephasing times of up to 500 ns and a gate quality factor that exceeds 10.

## Abstract (web summary)

A dispersive sensing technique, termed the radiofrequency electron cascade, can perform singlet-triplet readout of two exchange-coupled electron spins in a natural silicon planar metal–oxide–semiconductor quantum-dot array.

## Main

Silicon-based quantum processors have been used to demonstrate high-fidelity qubit initialization^1,2^, measurement^3–5^, and single-^6,7^ and two-qubit control^8–12^ in small-scale devices of up to two qubits, with fidelities exceeding the 99% threshold required to implement quantum error correction^13^. Electron spin qubits in quantum dots (QDs) have also been used for simple instances of quantum error correction in a three-qubit array^2^ and for the operation of a six-qubit processor^14^. Notably, such silicon-based quantum processors can be fabricated using industrial manufacturing techniques and integrated with cryogenic electronics^15,16^, thus offering a promising route to scaled quantum computing^17^.

Spin qubits based on silicon metal–oxide–semiconductor (MOS) technology^18,19^ and Si/SiGe heterostructures^10,14,20^ are of interest for industrial manufacturing. The MOS approach shares similarities with modern silicon field-effect transistor manufacturing, which can be used to form MOS QDs in both planar devices^11,18,19,21–23^ and etched silicon structures, such as nanowires^4,24,25^ and fin field-effect transistors^26,27^. Although the latter constrains the qubit topology to 2 × *N* bilinear QD arrays, the former offers easier scalability towards two-dimensional QD arrays^28^, which are essential for implementing quantum error-correcting codes, such as the surface code^13^. Single-qubit performance in MOS devices fabricated using semiconductor manufacturing lines has been demonstrated^24,27^, but quantum processors require two-qubit interactions to operate.

To enable further scaling, methods that simplify the readout infrastructure are required. The current standard for sensing—the radiofrequency (rf) single-electron transistor—provides high-fidelity readout^3,5^ at the cost of occupying substantial space on the qubit chip, which limits qubit connectivity. Fast and compact dispersive rf measurement techniques^29–31^ reduce the readout footprint. However, in situ dispersive readout metrics have seen limited progress in recent years^30^.

In this Article, we describe an in situ dispersive sensing mechanism, termed the rf electron cascade, that offers high spin qubit readout sensitivity and is demonstrated here in a planar silicon double quantum dot (DQD) device. With the readout method, we achieve a minimum integration time of 7.6 ± 0.2 μs, and using a physical model^32^, we calculate a 67 ± 1% singlet-triplet readout fidelity limited by spin relaxation. The measurement rate reported here represents an improvement of over two orders of magnitude compared with state-of-the-art results on dispersive readout in planar MOS devices^30^. We also demonstrate control of an exchange-mediated coherent interaction, which forms the basis for a $\sqrt{{\rm{SWAP}}}$ gate between two spin qubits.

## rf electron-cascade readout

Reading out spin qubits within semiconductor QDs typically involves mapping a spin state of interest onto a charge state of one or more QDs^33,34^, which can then be detected using a variety of charge-sensing methods^35^. For example, the Pauli spin blockade (PSB) can be used to map the singlet and triplet states of a pair of spin qubits onto two different charge configurations of a DQD (for example (1, 1) or (0, 2)), which are then detected by a single-electron transistor^36^ or single-electron box^4^. In situ dispersive readout of a DQD combines these into a single step, using the PSB to directly distinguish between singlet and triplet states through their difference in the alternating current (a.c.) polarizability^37^. This difference in polarizability is detected by incorporating the DQD into an rf tank circuit and measuring changes in the reflected rf signal. However, in situ dispersive readout has suffered from low sensitivity in planar MOS silicon quantum devices due to the relatively low gate lever arms^30^. To improve the sensitivity of this technique, we introduce a third QD coupled to a charge reservoir, which acts as an amplifier in measuring the a.c. polarizability of the DQD. Instead of measuring the usual single-electron a.c. generated by cyclic tunnelling between the two-spin singlet states of the DQD^35^, we leverage the synchronized single-electron a.c. current at the third dot-reservoir system generated as a consequence of the strong capacitive coupling to the DQD. Our approach offers the benefit of charge-enhancement techniques such as latching^38^, direct current (d.c.) cascading^39^ and spin-polarized single-electron boxes^25^ while retaining the non-demolition nature of in situ dispersive readout methods^40^.

**Fig. 1:**
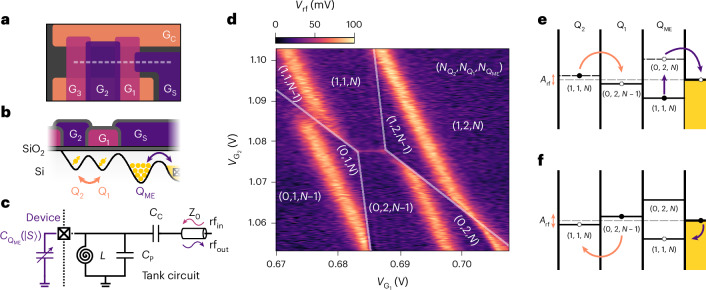
rf-driven electron cascade. **a**,**b**, Schematics of the top view (**a**) and cross section (**b**) (white dashed line in **a**) of the QD array. Gates G_1_ and G_2_ define the QDs Q_1_ and Q_2_, which are tuned to the two-electron occupancy. The DQD is capacitively coupled to dot Q_ME_, which is occupied by many electrons and is controlled by gate G_S_. Arrows indicate single-electron tunnelling events. **c**, Schematic of the rf resonator bonded to the ohmic contact of the device, including an equivalent circuit representation of the QD array as a spin-dependent variable capacitor ${C}_{{{\rm{Q}}}_{\mathrm{ME}}}(\left|\mathrm{S}\right\rangle )$. The resonator is formed by an off-chip superconducting spiral inductor *L* = 136 nH arranged in parallel with the parasitic capacitance *C*_P_ = 0.4 pF of the assembly. Connected to the transmission line Z_0_ via a coupling capacitor *C*_C_ = 0.1 pF, rf_in(out)_ represents the incident (reflected) rf signal on the resonator. **d**, Charge stability diagram of the DQD as a function of gate voltages ${V}_{{{\rm{G}}}_{1}}$ and ${V}_{{{\rm{G}}}_{2}}$. *V*_rf_ denotes the demodulated rf voltage. **e**,**f**, Schematic representations of the cascade process in which an rf excitation with amplitude *A*_rf_ synchronously drives charge transitions within the QD array. The reservoir is shown as the shaded region, and the dashed lines mark its Fermi level. **e**, A change in the charge occupation from (${N}_{{{\rm{Q}}}_{2}}$, ${N}_{{{\rm{Q}}}_{1}}$, ${N}_{{{\rm{Q}}}_{\mathrm{ME}}}$) = (1, 1, *N*) to (0, 2, *N* − 1) raises the electrochemical potential of the Q_ME_ above the Fermi level, causing one electron to synchronously escape to the reservoir. **f**, When the DQD is driven back to (1, 1, *N*), an electron tunnels back from the reservoir to Q_ME_.

We use a device based on planar silicon MOS technology with an overlapping gate design^41^ (Fig. 1a,b, Methods and Supplementary Section 1). QDs Q_1_ and Q_2_ form a DQD, which we tune to hold two electrons, while Q_2_ is capacitively coupled to a multi-electron QD (Q_ME_) that can exchange electrons with a charge reservoir. To measure the charge state of the system, we connect a superconducting spiral inductor to the reservoir to form an LC resonator (Fig. 1c). At voltages where the charge in Q_ME_ is bistable, cyclic tunnelling generated by the small rf signal supplied to the resonator produces a change in capacitance that can be detected as a change in the phase response Δ*Φ* or demodulated voltage *V*_rf_ using homodyne techniques^35^. Lines in the gate voltage space showing the Q_ME_ charge bistability are shifted when they intersect charge transitions of the DQD, as shown in Fig. 1d. Such shifts form the basis of dispersive charge-sensing measurements^4,42^, which we do not exploit here.

Instead, we focus on the directly observable a.c. signal in the region of the gate voltage space where charge transitions can occur between Q_1_ and Q_2_. We ascribe this signal to a two-electron charge cascade effect driven by the rf excitation, which we explain using the diagrams in Fig. 1e,f. Consider an rf cycle in which the system starts in the occupation configuration $({N}_{{{\rm{Q}}}_{1}},{N}_{{{\rm{Q}}}_{2}},{N}_{{{\rm{Q}}}_{\mathrm{ME}}})=(1,1,N)$. Owing to the strong capacitive coupling between Q_2_ and Q_ME_, the rf excitation that drives the DQD from the (1, 1) to the (0, 2) state synchronously forces an electron out of Q_ME_ in a cascaded manner, leading to (0, 2, *N* − 1). The second half of the rf cycle then reverses the process. Overall, the rf cascade measures the polarizability of the DQD system, as for in situ dispersive readout measurements^30,31^, but with the substantial advantage that the induced charge can be much greater, resulting in a dispersive measurement with greater sensitivity (see Supplementary Section 2 for more discussion on the requirements for rf electron-cascade readout).

**Fig. 2:**
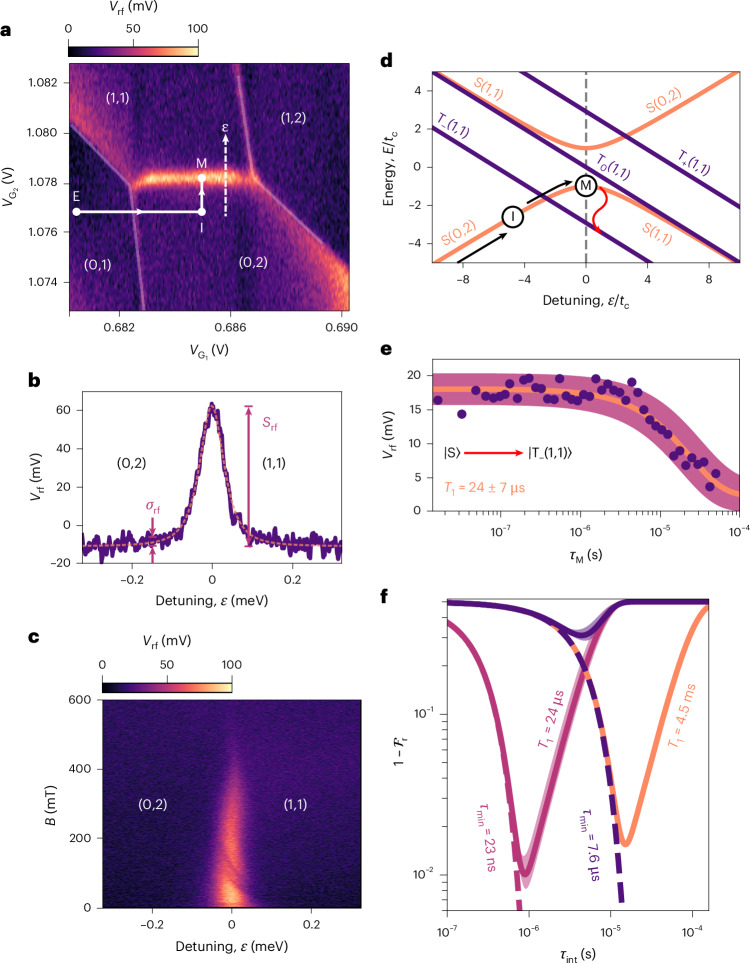
rf-cascade singlet-triplet readout. **a**, Charge stability diagram of the DQD around the (1, 1)–(0, 2) interdot charge transition, with a voltage pulse sequence (white arrows) and detuning axis *ε* overlaid. *V*_rf_ denotes the demodulated rf voltage. **b**, *V*_rf_ as a function of detuning across the interdot charge transition around *ε* = 0, fitted with equation (4) (dashed orange line). *S*_rf_ and *σ*_rf_ represent the signal amplitude and standard deviation, respectively. **c**, Magneto-spectroscopy of the interdot charge transition as a function of applied magnetic field *B* and *ε*. **d**, Energy diagram showing the dependence of the two-electron spin states, singlet $\left|{\rm{S}}\right\rangle$ and triplet $\left|{\rm{T}}\right\rangle$, as a function of *ε* with respect to the interdot tunnel-coupling *t*_c_. The pulse sequence depicts the initialization (I) of $\left|{\rm{S}}{(0,\; 2)}\right\rangle$ via an adiabatic ramp from the (0, 1) empty (E) state, followed by a non-adiabatic pulse to *ε* = 0 for measurement (M). **e**, *V*_rf_ as a function of wait time *τ*_M_ at *ε* = 0 before measurement, following the pulse sequence depicted in **a** and **d**. The fitted line indicates a $\left|\mathrm{S}\right\rangle$ to $\left|{\rm{T}}_{-}(1,1)\right\rangle$ relaxation time *T*_1_ = 24 μs. **f**, Calculated readout infidelity $1-{{\mathcal{F}}}_{{\rm{r}}}$ as a function of integration time *τ*_int_ as described by equation (7). Solid lines depict the fidelity with a limited relaxation time *T*_1_, whereas dashed lines depict it for *T*_1_ = *∞*. Parameters used from left to right: ${\tau }_{\min }$ = {23 ns, 7.6 μs, 7.6 μs} and *T*_1_ = {24 μs, 24 μs, 4.5 ms}. In both **e** and **f**, shaded areas indicate the propagated error from the ±1 standard deviation of the fitted parameters.

**Extended Data Fig. 1:**
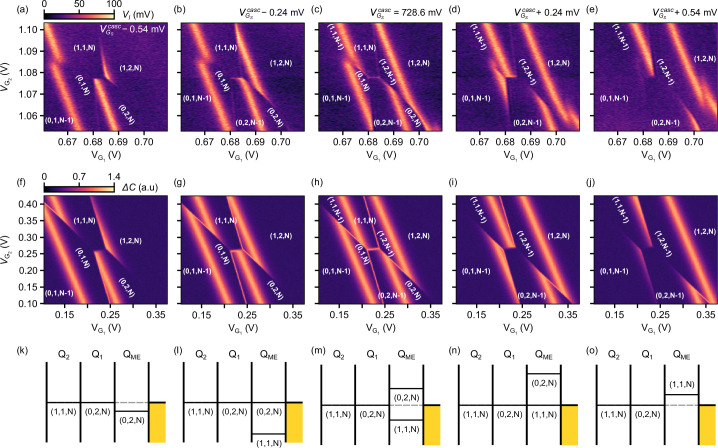
Tuning the radiofrequency driven electron cascade. **a-e**, Charge stability diagrams as a function of the DQD gate voltages ${V}_{{{\rm{G}}}_{1}}-{V}_{{{\rm{G}}}_{2}}$. The multi-electron QD bias ${V}_{{G}_{{\rm{S}}}}$ is varied in each panel, defined with respect to ${V}_{{{\rm{G}}}_{{\rm{S}}}}^{{\rm{casc}}}$, the bias where the conditions for cascade are observed, shown in panel **c**. This is the bias utilized in the main text of the manuscript. Similar data for additional devices can be found in Supplementary Section 2. **f-j**, Simulated charge stability diagrams using the methods described in Supplementary Section 4. **k-o** Schematic depiction of the electrochemical levels for the corresponding bias conditions, arranged column-wise.

Specifically, we find the power signal is amplified in the cascade approach compared with direct dispersive readout, by a factor

1 $$A={\left(1+\frac{1-{\alpha }_{{\rm{R}},{\rm{ME}}}}{{\alpha }_{{\rm{R}},2}-{\alpha }_{{\rm{R}},1}}\right)}^{2} > 1,$$

where *α*_R,*j*_ represents the gate lever arm between the reservoir and QD *j*. In particular, the sensor detects not only the interdot gate polarization charge (*α*_R,1_ − *α*_R,2_)*e*, where e is the charge on an electron, but also the cascaded charge collected at the reservoir, (1 − *α*_R,ME_)*e* (see Supplementary Section 3 for a derivation). By comparing the measured signal-to-noise ratio (SNR) with and without the cascade effect, we find a lower bound for the power amplification factor, *A* ≥ (3.4 ± 0.1) × 10^3^ (+35.4 dB). The cascade SNR is extracted from the fit for the interdot charge transition shown in Fig. 2a,b (equations (4) and (5)). If the cascade is absent, the SNR is assumed to be upper bounded by ≤ 0.5, as there is no observable signal, as shown in Extended Data Fig. 1a,e. From the cascade SNR, we extract a minimum integration time of ${\tau }_{\min }=7.6\pm 0.2\,{\upmu}{\rm{s}}$ (equation (6)), which is an improvement of over two orders of magnitude compared with previous planar MOS demonstrations of in situ dispersive readout^30^.

We use the rf cascade to distinguish between the singlet and triplet states of the DQD via the PSB. The signature of the PSB can be observed by measuring the asymmetric disappearance of the interdot charge transitions as the applied magnetic field is increased^37^, as shown in Fig. 2c. At low magnetic field (*B* ≤ 200 mT), the system is free to oscillate between singlet states ($\left|\mathrm{S}(1,1)\right\rangle \leftrightarrow \left|\mathrm{S}(0,2)\right\rangle$) due to the action of the rf drive, yielding a signal in the rf response. However, at higher fields (*B* ≥ 200 mT), the polarized triplet $\left|\mathrm{T}_{-}(1,1)\right\rangle$ state becomes the ground state for *ε* ≥ 0 (Fig. 2d), which prevents a charge transition and results in the disappearance of the signal, initially for the region of the transition closer to the (1, 1) charge configuration. A quantum capacitance-based simulation of the data shown in Fig. 2c indicates an interdot tunnel-coupling of *t*_c_ = 2.4 GHz and electron temperature *T*_e_ = 50 mK (Supplementary Section 5). Figure 2e shows the decay from $\left|\mathrm{S}\right\rangle$ to $\left|\mathrm{T}_{-}(1,1)\right\rangle$ at *ε* = 0 for applied magnetic field *B* = 250 mT. The corresponding relaxation time *T*_1_ = 24 ± 7 μs, which is consistent with a study of a similar device, where a relaxation time of *T*_1_ = 10 μs was observed near zero detuning for a comparable tunnel-coupling (*t*_c_ ≥ 1.9 GHz)^43^. In that study, *T*_1_ was found to vary exponentially with *t*_c_, with relaxation times exceeding 100 ms for *t*_c_ < 1 GHz. Lowering *t*_c_ would also enhance the readout sensitivity, as the dispersive signal is maximized at 2*t*_c_ = *f*_rf_ (ref. ^44^), where *f*_rf_ is the resonator drive frequency, which we set to 512 MHz.

We assess the performance of the rf-driven electron cascade by calculating the readout fidelity ${{\mathcal{F}}}_{{\rm{r}}}$ as a function of integration time using equation (7)^32^. We calculate a relaxation-limited readout fidelity of ${{\mathcal{F}}}_{{\rm{r}}}=67\pm 1 \%$ based on the experimentally obtained ${\tau }_{{\mathrm{min}}}=7.6\,\upmu {\mathrm{s}}$ and integration time *τ*_int_ = 8 μs. The corresponding readout infidelity $1-{{\mathcal{F}}}_{{\rm{r}}}$ is depicted in Fig. 2f (dark purple line). This infidelity is comparable to previous in situ readout demonstrations but was achieved with integration times that are 1–2 orders of magnitude faster than those reported in previous silicon planar MOS and implanted donor systems^29,30^ (Supplementary Section 7). To achieve 99% fidelity, two strategies are indicated in Fig. 2f: (1) Reduce the minimum integration time to 24 ns (light purple line) by enhancing the SNR through resonator optimization, quantum-limited amplification or both^35,45^. (2) Extend the relaxation time of the system, which for the *T*_1_ = 4.5 ms reported in ref. ^30^ yields the orange line.

We highlight that, unlike other charge-enhancement techniques^25,38,39^, the rf electron cascade retains the non-demolition nature of in situ dispersive readout measurements, as the DQD system remains in an eigenstate after a measurement is performed^1,46^. We note that the rf excitation is continuously applied for all measurements in this Article, and we discuss the potential impact of this in the section ‘Echo sequence’.

## Characterizing the spin–orbit coupling

**Fig. 3:**
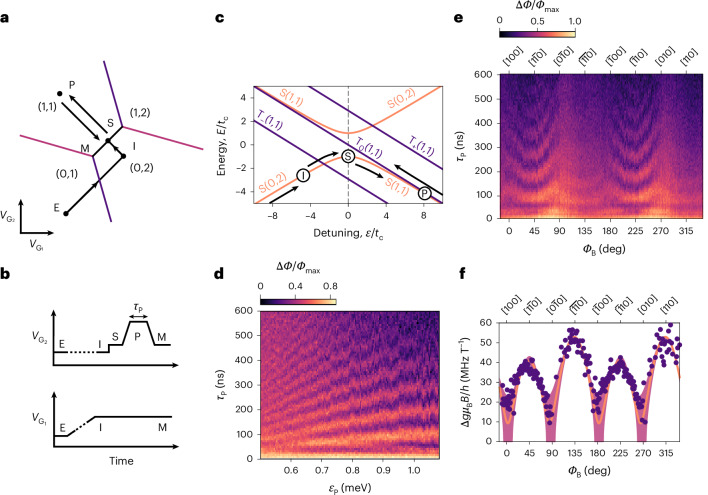
Measurement of the SOI. **a**, Schematic of the DQD charge stability diagram around the (1, 1)–(0, 2) interdot charge transition with voltage pulse sequence EISPM overlaid (see Methods). **b**, The pulse sequence shown as a function of time. The dashed lines indicate longer durations. **c**, Energy diagram showing the dependence of the two-electron spin states, singlet $\left|\mathrm{S}\right\rangle$ and triplet $\left|\mathrm{T}\right\rangle$, as a function of detuning *ε* with respect to the interdot tunnel-coupling *t*_c_. **d**, $\left|\mathrm{S}\right\rangle$–$\left|\mathrm{T}_{0}\right\rangle$ oscillations as a function of duration *τ*_P_ and detuning at point P, *ϵ*_P_, in the pulse sequence (applied magnetic field *B* = 250 mT and in-plane magnetic field orientation *ϕ*_B_ = 235°). **e**, $\left|\mathrm{S}\right\rangle$–$\left|\mathrm{T}_{0}\right\rangle$ oscillation frequency dependence as a function of *ϕ*_B_, measured as changes in rf phase with respect to a global maximal phase *Φ*_max_ (a fixed detuning of *ε*_P_ = 0.926 meV is used). The oscillation frequencies were obtained from a Fourier analysis. **f**, The SOI component of the extracted frequencies, Δ*gμ*_B_*B*/*h*, was fitted (line) using the model described in equation (2). Here Δ*g* is the difference in *g* factors between QDs, *μ*_B_ is the Bohr magneton, *B* is the applied magnetic field and *h* is Planck’s constant. The shaded area shows the propagated error from the ±1 standard deviation of the fitted parameters.

Having established a method that distinguishes between singlet and triplet spin states, our goal is to prepare and coherently control spin states of the DQD through voltage pulses along the detuning axis, *ϵ*. Such pulses bring the DQD: (1) from the (0, 2) charge configuration in which a singlet is prepared, (2) into the (1, 1) region where the electron spins are spatially separated between QDs and may evolve, and (3) back to an intermediate point where they can be measured (Fig. 3a–c). Deep in the (1, 1) region, the spin basis states are predominantly $\left|\uparrow \downarrow \right\rangle$ and $\left|\downarrow \uparrow \right\rangle$. Under an adiabatic ramp to *ϵ* = 0 for readout, these two states map onto the $\left|\mathrm{T}_{0}(1,1)\right\rangle$ and $\left|\mathrm{S}(0,2)\right\rangle$ states, respectively. The basis states are separated in energy by $h\varOmega =\sqrt{J{(\varepsilon )}^{2}+\Delta {E}_{{\rm{z}}}^{2}}$, where we include the kinetic exchange interaction *J*(*ε*) and the Zeeman energy difference between electrons in each dot Δ*E*_z_. The spin detuning Δ*E*_z_ = Δ*gμ*_B_*B* + *gμ*_B_Δ*B*_HF_ (where *μ*_B_ is the Bohr magneton and *h* is Planck’s constant) contains two main contributions: (1) the difference in *g* factor between QDs $\Delta g=\left|{g}_{2}-{g}_{1}\right|$ arising from variations in the spin–orbit interaction (SOI) near the Si/SiO_2_ interface^22,47,48^ and (2) the difference in the effective ^29^Si nuclear magnetic field experienced by each QD, Δ*B*_HF_. The random fluctuations in the effective magnetic field experienced by each electron in the DQD can be described by a normal distribution with mean of 0 (given the negligible spin polarization) and standard deviation *σ*_HF_ = 30 ± 4 μT, as we shall see later. This value corresponds to a hyperfine energy strength of 3.4 ± 0.4 neV, which aligns well with other reports for natural silicon^20,49–51^.

In previous work, the spin detuning Δ*E*_z_ was leveraged to drive oscillations between the $\left|\mathrm{S}\right\rangle$ and $\left|\mathrm{T}_{0}\right\rangle$ states^22,23,51,52^. At an applied magnetic field *B* = 250 mT, we observe similar oscillations using the pulse sequence presented in Fig. 3a–c. We start in the (0, 1) configuration by emptying dot Q_1_, then initialize the $\left|\mathrm{S}(0,2)\right\rangle$ state via an adiabatic ramp across the (0, 1)–(0, 2) charge transition. A fast non-adiabatic pulse to *ε*_P_ in the (1, 1) region leads to oscillations between $\left|\mathrm{S}\right\rangle$ and $\left|\mathrm{T}_{0}\right\rangle$ over the dwell time *τ*_P_. The final state is then measured dispersively using a non-adiabatic pulse back to the (1, 1)–(0, 2) charge transition at *ε* = 0 for readout. The $\left|\mathrm{S}\right\rangle$ to $\left|\mathrm{T}_{0}\right\rangle$ oscillations shown in Fig. 3d provide a direct measurement of *Ω*. For *ε*_P_ ≳ 0.9 meV, the dependence of the oscillation frequency on detuning is substantially reduced, indicating that, in this region, the Δ*E*_z_ term dominates (*J*(*ϵ*) ≤ Δ*E*_z_), as Δ*g* is only weakly dependent on the detuning $\left(\partial \Delta g/\partial \varepsilon \approx 0\right)$^23,52^. As we shall see later, at the deepest detuning (*ε*_P_ = 1.054 meV), we find *J*/Δ*E*_z_ = 0.5 ± 0.3.

The strength of the SOI means that the Δ*g* term depends on Rashba and Dresselhaus spin–orbit couplings. The SOI (and, hence, Δ*g*) can be tuned by varying the electrostatic confinement perpendicular to the interface and the transverse magnetic field^22,47^. We vary the orientation of the in-plane magnetic field and observe changes in the $\left|\mathrm{S}\right\rangle$–$\left|\mathrm{T}_{0}\right\rangle$ oscillation frequency, as shown in Fig. 3e,f. We fit the variation in *Ω* as a function of the angle *ϕ*_B_ between the [100] crystal axis and the applied (in-plane) magnetic field^22^,

2 $$\Delta {E}_{{\rm{z}}}/h=\sqrt{{\left(\left|B\right|\,\left|\Delta \alpha -\Delta \beta \,\sin (2{\phi }_{{\rm{B}}})\right|\right)}^{2}+{\left(g{\mu }_{{\rm{B}}}{\sigma }_{\mathrm{HF}}/h\right)}^{2}}.$$

The Rashba and Dresselhaus SOI terms are, respectively, captured by Δ*α* and Δ*β*. We find $\Delta \alpha =6.2_{-1.5}^{+1.6}$ MHz T^−1^ and $\Delta \beta =45_{-7}^{+5}$ MHz T^−1^, which are larger than other reported values^22,48,53^ and could be partially influenced by the large asymmetry in the gate biasing conditions. The fit assumes that for the fixed detuning *ε*_P_ = 0.926 meV used here, the residual exchange interaction *J*(*ε*_P_)/*h* = 6.3 ± 1.9 MHz is independent of the in-plane magnetic field orientation. In the following sections we operate at an in-plane magnetic field direction near the $\left[1\bar{1}0\right]$ direction at *ϕ*_B_ = 55° (235°). Overall, this section expands the recent studies of the SOI in isotopically purified ^28^Si MOS nanostructures^22,23,48,53^ to natural silicon, where the non-negligible effect of the Overhauser field needs to be taken into account.

## Exchange control

**Fig. 4:**
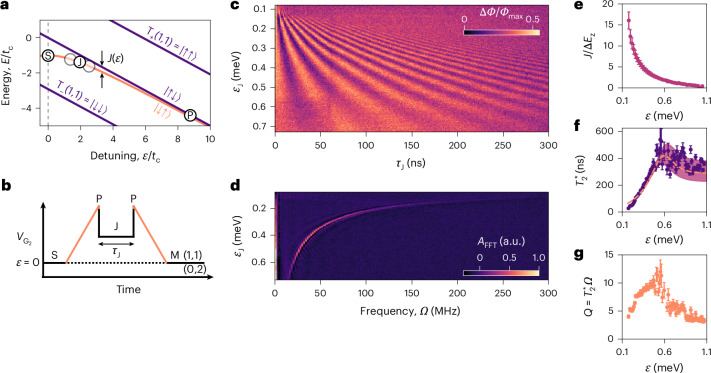
Exchange control in a natural-Si DQD device. **a**, Energy diagram depicting two-electron spin states in the (1, 1) detuning *ε* regime, with pulse sequence steps overlaid. Each axis is plotted with respect to the interdot tunnel-coupling *t*_c_. **b**, Detuning pulse sequence including initialization (P) to the $\left|\downarrow \uparrow \right\rangle$ state via a semi-adiabatic ramp (orange), followed by a non-adiabatic pulse (J) to near zero detuning to increase the exchange coupling *J*(*ε*) for duration *τ*_J_. **c**, Exchange-driven oscillations between the $\left|\downarrow \uparrow \right\rangle$ and $\left|\uparrow \downarrow \right\rangle$ states. The measured rf-phase response is proportional to the singlet probability. **d**, The corresponding fast Fourier transform amplitude *A*_FFT_. **e**, Ratio between exchange coupling strength *J*(*ε*) and dot-to-dot Zeeman energy difference Δ*E*_z_. **f**, Dephasing time ${T}_{2}^{* }$ (purple dots) extracted from the decay of the exchange oscillations and fitted with ${T}_{2}^{* }=\sqrt{2}\hslash /{\rm{\delta }}\left(h\varOmega \right)$ (orange dashed line)^49,54^. The shaded area shows the propagated error from the ±1 standard deviation of the fitted parameters. **g**, Qubit quality factor $Q={T}_{2}^{* }\varOmega$. In **e** and **g**, each data point was extracted as a fitted parameter at a fixed detuning *ε*_J_ point of **c**. The dataset for each fit consists of around 300 points obtained from over 64 × 10^3^ averages. Each error bar represents the standard deviation of a fitted parameter.

We implement exchange control using the sequence depicted in Fig. 4a,b, where the $\left|\downarrow \uparrow \right\rangle$ state is initialized via a ramp from *ε* = 0 into the (1, 1) configuration, which is adiabatic with respect to *E*_z_ (ref. ^20^). A fast non-adiabatic pulse towards zero detuning increases the exchange coupling, driving oscillations between the $\left|\downarrow \uparrow \right\rangle$ and $\left|\uparrow \downarrow \right\rangle$ states at frequency *Ω*(*ε*), as observed in Fig. 4c. The final state after some evolution time *τ*_J_ is projected to $\left|\mathrm{S}\right\rangle$ or $\left|\mathrm{T}_{0}\right\rangle$ for readout. The Fourier transform of the exchange oscillations (Fig. 4d) reveals a single peak with increasing frequency as the detuning is reduced, indicating the purity of the oscillations and the enhanced exchange strength at lower detuning. From this, we observe that the exchange coupling is tunable over a range of *J* = 5–122 MHz.

To quantify the properties of these rotations, we combine the results of the exchange oscillations in Fig. 4c and the $\left|\mathrm{S}\right\rangle$–$\left|\mathrm{T}_{0}\right\rangle$ oscillations in Fig. 3d, to extract the ratio *J*/Δ*E*_z_ and the intrinsic coherence time ${T}_{2}^{* }$ over a wide range of detunings (Fig. 4e,f). We extract ${T}_{2}^{* }$ by fitting the oscillations at each detuning point with a Gaussian decay envelope of the form exp$[-{(\tau /{T}_{2}^{* })}^{2}]$, and then obtain Δ*E*_z_ = 9.6 ± 1.2 MHz from the fit to the expression

3 $$\frac{1}{{T}_{2}^{* }}=\frac{1}{\sqrt{2}\hslash }\sqrt{{\left(\frac{J}{h\varOmega }\frac{{\rm{d}}J}{{\rm{d}}\varepsilon }{\rm{\delta }}{\varepsilon }_{\mathrm{rms}}\right)}^{2}+{\left(\frac{\Delta {E}_{z}}{h\varOmega }\delta \Delta {E}_{{\rm{z}},\mathrm{rms}}\right)}^{2}},$$

where δ*ε*_rms_ and δΔ*E*_z,rms_ refer to the root mean square (r.m.s.) of the fluctuations in *ε* and Δ*E*_z_ (refs. ^49,54^).

The extracted *J*/Δ*E*_*z*_ ratio is shown in Fig. 4e. It reduces for increasing *ε* to a minimum value of 0.5 ± 0.3 at *ε* = 1.054 meV (beyond this point, we cease to observe oscillations). This non-zero minimum shows that there remains a residual exchange that cannot be fully turned off, which should be taken into account when designing two-qubit exchange gates.

From the ${T}_{2}^{* }$ data shown in Fig. 4f, we observe a rapid increase in coherence as the detuning increases from zero, indicative of a low δ*ε*_rms_. The extracted δ*ε*_rms_ = 5.4 ± 0.1 μeV, obtained over a measurement time of 0.3 h per trace, is comparable with that of other MOS devices^21,22,51^ and is consistent with the previously reported low charge noise levels achieved for samples using this 300-mm process^55^, which are comparable to those observed in SiGe heterostructures^56^ (Methods). As the detuning increases further, when *J* < Δ*E*_z_, we observe that the noise in Δ*E*_*z*_ dominates (due to ^29^Si nuclear spins), leading to a relatively constant ${T}_{2}^{* }$. From this saturation value of ${T}_{2}^{* }=0.28\pm 0.04\,\upmu {\mathrm{s}}$, we extract ${\sigma }_{\mathrm{HF}}=\sqrt{2}{\rm{\pi }}\delta \Delta {E}_{{\mathrm{z}},{\mathrm{rms}}}/(g{\upmu }_{{\rm{B}}})=30\pm 4\,\upmu {\mathrm{T}}$. Note that we assume the Zeeman energy fluctuations are dominated by the Overhauser field rather than noise in the *g* factor difference.

The entangling two-qubit gate achieved between the spin qubits under the exchange interaction depends on the ratio *J*/Δ*E*_z_, tending to a $\sqrt{\,{\rm{SWAP}}}$ operation as *J* ≫ Δ*E*_z_ or a C phase when *J* ≪ Δ*E*_z_, although any gate within this set parameterized by *J*/Δ*E*_z_ can be used as the building block for a quantum error-correcting code such, as the surface code^57^. Defining the qubit quality factor as $Q={T}_{2}^{* }\varOmega$ (the number of periods before the amplitude of oscillations decays by 1/*e*), we find *Q* ≥ 10 in the region *J*/Δ*E*_z_ = 2.1–3.2, on par with previous reports across a range of semiconductor platforms^22,49,50,52,54^ (Supplementary Section 8). This provides an upper bound estimate on the achievable two-qubit gate fidelity using the approximation ${\mathcal{F}}\approx 1-1/4Q\le 98 \%$ (ref. ^58^). To implement error-correctable two-qubit gates, this fidelity would need to surpass 99% (ref. ^13^), which could be achieved using isotopically enriched silicon. In the section ‘Echo sequence’, we extend the coherence time using spin refocusing techniques.

## Echo sequence

**Fig. 5:**
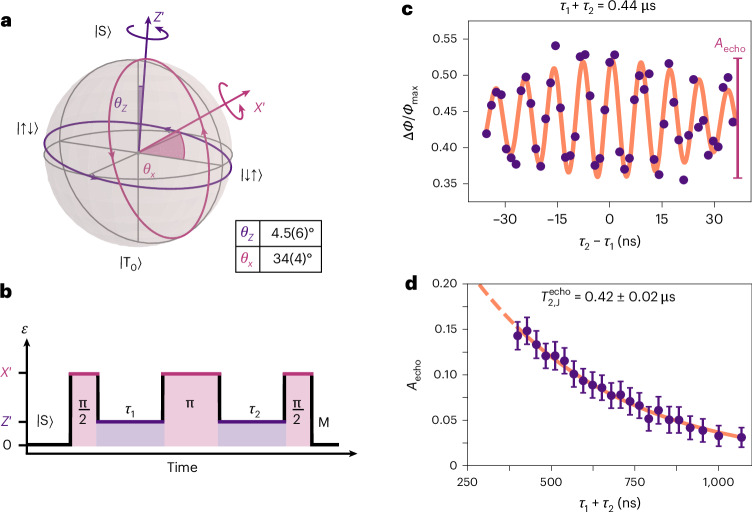
Echo sequence. **a**, Bloch sphere representation of the odd-parity two-spin subspace, indicating the rotation axes, ${\widehat{Z}}^{{\prime} }$ and ${\widehat{X}}^{{\prime} }$ and their angular deviation (*θ*_*Z*_, *θ*_*X*_) from the nominal $\widehat{Z}$ axis defined by $\left|\mathrm{S}\right\rangle$ and $\left|\mathrm{T}_{0}\right\rangle$. **b**, Schematic of the exchange echo sequence. **c**, Echo signal data (purple dots) as a function of free evolution time difference *τ*_2_ − *τ*_1_, fitted with a decay envelope (orange line) with amplitude *A*_echo_. **d**, Echo amplitude as a function of total free evolution time *τ*_1_ + *τ*_2_ where *τ*_2_ = *τ*_1_. The data (purple dots) were fitted with an exponential decay (dashed orange line). Error bars indicate the standard deviation of the fitted *A*_echo_ data.

Dephasing of the two-electron spin state due to low-frequency electric or magnetic noise can be corrected using refocusing pulses. We implement an echo sequence by combining periods of evolution at different detuning points to achieve rotations around the two axes ${Z}^{{\prime} }$ and ${X}^{{\prime} }$ shown in Fig. 5a. The specific sequence shown in Fig. 5b, termed the exchange echo, primarily reduces the impact of electric noise^22,54^.

In the exchange echo sequence, after initializing a $\left|\mathrm{S}(1,1)\right\rangle$ state and applying a ${X}_{{\rm{\pi }}/2}^{{\prime} }$ rotation, the two-electron system dephases under the effect of charge noise for a time *τ*_1_. The free evolution occurs at a detuning point where *J*/Δ*E*_z_ = 12.8 ± 1.6, for which we measured ${T}_{2}^{* }=43\pm 3$ ns (Fig. 4f). We then refocus the spins by applying a ${X}_{{\rm{\pi }}}^{{\prime} }$ rotation and let the system evolve for *τ*_2_ until a second ${X}_{{\rm{\pi }}/2}^{{\prime} }$ rotation maps the resulting state to the $\left|\mathrm{S}\right\rangle$–$\left|\mathrm{T}_{0}\right\rangle$ axis. We extract the amplitude of the echo by fitting the signal in the *τ*_2_ − *τ*_1_ domain (Fig. 5c), and plot its value as a function of total free evolution time *τ*_1_ + *τ*_2_. Figure 5d shows that the echo amplitude decays exponentially with total time (*τ*_2_ + *τ*_1_), yielding a characteristic ${T}_{2,{\rm{J}}}^{{\mathrm{echo}}}=0.42\pm 0.02\,\upmu {\mathrm{s}}$, which corresponds to an order of magnitude increase in the coherence time, like previous work with silicon devices^22,50^. From our fit to equation (3), we extract a magnetic-noise-limited ${T}_{2,\Delta {E}_{{\rm{z}}}}^{* }=3.3\,{\upmu}{\rm{s}}$ at this set point, indicating that sources other than magnetic noise limit ${T}_{2,{\rm{J}}}^{\,\mathrm{echo}}$. This finding is in agreement with previous reports, which show that ${T}_{2,{\rm{J}}}^{\mathrm{echo}}$ is an order of magnitude shorter than the magnetic noise limit^22,50^ (Supplementary Section 9). We speculate that residual high-frequency charge noise limits the echo coherence^6^. This noise may include factors such as the effect of the rf readout excitation, which is active throughout the control sequence. We estimate the upper bound on detuning fluctuations associated with this rf excitation to be ${\rm{\delta }}{\varepsilon }_{{\mathrm{rms}}}^{{\mathrm{rf}}}\le 2.7\,\upmu {\mathrm{eV}}$ (equation (8)). However, this limitation is not intrinsic to the the readout scheme itself, as the effect can be effectively mitigated by implementing a pulsed readout protocol. In this approach, the rf drive is deactivated during the control sequence and activated only during the readout phase.

## Conclusions

**Fig. 6:**
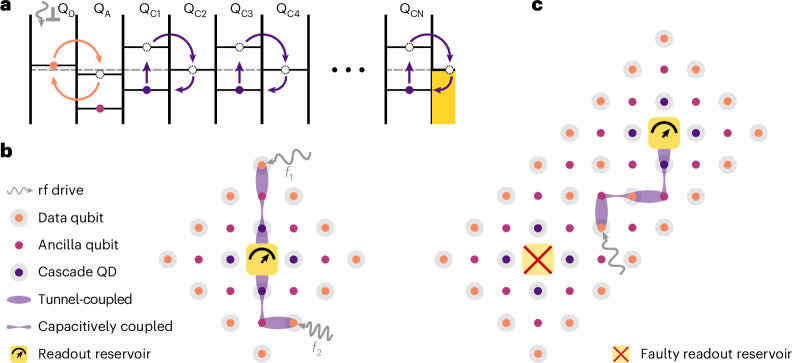
Extending the rf-cascade readout to two-dimensional arrays. **a**, Schematic representation of the cascade process extended to an arbitrarily long one-dimensional array. Readout of the PSB is achieved by tunnel-coupling the data qubit Q_D_ (orange) and ancilla qubit Q_A_ (light purple). The resulting DQD is capacitively coupled to a chain of DQDs tuned into the cascade configuration (${{\rm{Q}}}_{{\rm{CN}}}$) (dark purple), which enables in situ dispersive readout at an arbitrary distance. **b**, Schematic of a two-dimensional array of unit cells centred around a readout reservoir (yellow) and connected to an rf reflectometry readout apparatus. Simultaneous multiplexed readout is achieved by applying distinct rf frequencies *f*_1_ and *f*_2_ to separate electrostatic gates, each controlling data qubit QDs driving distinct cascaded charge transitions. **c**, Repetition of the unit cell highlighting the robustness of the scheme to points of failure, such as a faulty readout reservoir, by rerouting the readout cascade chain to the nearest reservoir.

We have described rf electron-cascade readout, a high-gain in situ dispersive readout technique that we demonstrated here in a planar MOS device. The rf-driven electron cascade could be extended to larger arrays for distant readout, which would eliminate the need for swap-based schemes that rely on shuttling information to a sensor^59^. Such an extension builds upon existing schemes for two-dimensional grids using data and ancilla qubits (Fig. 6)^4,13^. We assume that the tunnel barriers between each QD can be precisely controlled to enable optimal readout fidelity through tuning *t*_c_ (ref. ^5^).

Figure 6a illustrates the readout protocol in a simplified one-dimensional array. To readout the data qubit Q_D_ at the periphery of the unit cell, the data qubit is tunnel-coupled to a neighbouring ancilla qubit Q_A_. The ancilla qubit, in turn, is capacitively coupled to a chain of DQDs configured in the cascade configuration and eventually connected to a reservoir. An rf drive applied to the ancilla-data DQD initiates the cascade, which propagates along the chain to a readout reservoir. This scheme uniquely enables the simultaneous readout of distant data qubits through frequency multiplexing at the readout tank circuit. By driving several cascade chains at distinct frequencies, several distant qubits can be read out simultaneously (Fig. 6b), in contrast to earlier schemes^39^. Furthermore, combining unit cells into a dense, scalable two-dimensional grid allows the readout to share resources, resulting in the system being resilient to failure points, such as faulty QDs or reservoirs (Fig. 6c).

Furthermore, we have demonstrated exchange control, which forms the basis for two-qubit gates between spin qubits, in a natural planar silicon MOS DQD device. The measured detuning noise is comparable with that of other planar MOSs^55^, and the ${T}_{2}^{* }$ is relatively long for a natural silicon device^58^. These results are consistent with reports on devices from the same 300-mm fabrication line^55^, indicating that process quality is a contributing factor. Follow-up studies could include measurements in isotopically enriched Si samples^12^ (Supplementary Section 10). Further work could also extend exchange control to larger spin qubit arrays by adapting dedicated gates^11,19^ to primarily control the exchange strength over a wider range. This could enable symmetric exchange pulses and reduce *J*/Δ*E*_z_ to well below 1, which should lead to an overall reduction in sensitivity to charge noise.

## Methods

### Fabrication details

The device measured in this study was fabricated on a natural silicon 300-mm wafer, with three 30-nm-thick in situ n^+^ phosphorus-doped polycrystalline silicon gate layers formed with a wafer-level electron-beam patterning process^41,55^. We used a high-resistivity (>3 kΩ cm^−1^) p-type Si wafer. First, an 8-nm-thick, high-quality SiO_2_ layer was grown thermally to minimize the density of defects in the oxide and at the interface. Then, we subsequently patterned the gate layers using litho-etch processes and electrically isolated them from one another with a 5-nm-thick blocking high-temperature-deposited SiO_2_ layer (ref. ^41^). We employed the first layer of gates (closest to the silicon substrate) to provide in-plane lateral confinement in the direction perpendicular to the DQD axis. We used the second layer of gates (G_2_ in this case) to form and control primarily QD Q_2_. Finally, we used the third gate layer to form and control QD Q_1_ via G_1_ and both the multi-electron QD Q_ME_ and reservoir via G_S_, as shown in Fig. 1a,b. Supplementary Section 1 includes scanning and transmission electron microscopy images of structures like the QD array.

### Measurement set-up

We performed the measurements at the base temperature of a dilution refrigerator (*T* ≈ 10 mK). We sent low-frequency signals through cryogenic low-pass filters with a cutoff frequency of 65 kHz, while we applied pulsed signals through attenuated coaxial lines. Both signals were combined through bias T’s at the level of the sample printed circuit board. The printed circuit board was made from 0.8-mm-thick RO4003C with an immersion silver finish. For readout, we used rf reflectometry applied on the ohmic contact of the device. We sent rf signals through attenuated coaxial lines to an LC resonator on the printed circuit board and arranged in a parallel configuration. The resonator had a coupling capacitor (*C*_c_), a 100-nm-thick NbTi superconducting spiral inductor (*L*) and the parasitic capacitance to ground (*C*_p_), as shown in Fig. 1c. We drove the resonator at 512.25 MHz, which was the frequency of the system when G_S_ was well above the threshold. The reflected rf signal was then amplified at 4 K and room temperature, followed by quadrature demodulation, from which the amplitude and phase of the reflected signal were obtained (homodyne detection).

### rf-cascade readout performance

The SNR of the interdot charge transition shown in Fig. 2b was determined by fitting the signal to an expression proportional to the quantum capacitance, which is proportional to ∂^2^*E*/∂*ε*^2^ (refs. ^35,37^),

4 $${V}_{{\rm{I}},{\rm{Q}}}\propto {V}_{{\rm{I}},{\rm{Q}}}^{0}-{S}_{{\rm{I}},{\rm{Q}}}\frac{{(2{t}_{{\rm{c}}})}^{3}}{{\left({(\varepsilon -{\varepsilon }_{0})}^{2}+{(2{t}_{{\rm{c}}})}^{2}\right)}^{3/2}},$$

where *t*_c_ is the tunnel-coupling, *ε*_0_ is the detuning value of the centre of the peak, and *S*_I,Q_ and ${V}_{{\rm{I}},{\rm{Q}}}^{0}$ are the voltage signal and voltage offset of the phase and quadrature components of the measured rf response, respectively. The power SNR was determined by combining the measured in-phase and quadrature voltage signal components of the reflected rf signal,

5 $$\mathrm{SNR}=\frac{{S}_{{\rm{I}}}^{2}+{S}_{{\rm{Q}}}^{2}}{0.5({\sigma }_{{\rm{I}}}^{2}+{\sigma }_{{\rm{Q}}}^{2})},$$

where *S*_rf,I(Q)_ and *σ*_rf,I(Q)_ are indicated in Fig. 2b and represent the signal and the standard deviation in the background signal (away from zero detuning), respectively. Here we assume a white noise spectrum dominated by the first cryogenic amplification stage^60^. In the measurements acquired throughout the text, the applied rf power was *P*_rf_ = −88.5 dBm, corresponding to a power SNR = (1.7 ± 0.1) × 10^3^. The minimum integration time was determined using^35^

6 $${\tau }_{\min }=\frac{{N}_{{\rm{avg}}}}{2\Delta f\,{\rm{SNR}}}$$

where *N*_avg_ = 4,000 is the number of averages used in the measurement, and Δ*f* = 1.53*f*_LPF_ is the measurement bandwidth set by the low-pass filter cutoff frequency *f*_LPF_ = 100 kHz. Together, these two parameters give the noise-equivalent integration time *τ*_NE_ = 13 ms for the measurement in Fig. 2b. Substituting the SNR into equation (6), we found the minimum integration time ${\tau }_{{\mathrm{min}}}=7.6\pm 0.2\,\upmu {\mathrm{s}}$. In combination with the relaxation time *T*_1_ extracted in Fig. 2e, the readout fidelity was calculated using the following expression^32^:

7 $${{\mathcal{F}}}_{{\rm{r}}}=\frac{1}{2}\left[1+\mathrm{erf}\left(\sqrt{\frac{{\tau }_{\mathrm{int}}}{8{\tau }_{\min }}}\right)\exp \left(-\frac{{\tau }_{\mathrm{int}}}{2{T}_{1}}\right)\right],$$

where *τ*_int_ is the integration time and erf(*x*) is the Gaussian error function. The corresponding readout infidelity $1-{{\mathcal{F}}}_{{\rm{r}}}$ is shown in Fig. 2f.

We define the detuning fluctuations associated with the rf excitation to be given by^35^

8 $$\delta {\varepsilon }_{{\rm{rms}}}^{{\rm{rf}}}=\frac{{\alpha }_{21}{V}_{{\rm{dev}}}}{\sqrt{2}},$$

where *α*_21_ = *α*_R,2_ − *α*_R,1_ and *V*_dev_ is the voltage excitation at the reservoir ohmic contact the from input rf signal *V*_in_ (ref. ^61^):

9 $${V}_{\mathrm{dev}}=\frac{2{C}_{{\rm{C}}}{Q}_{{\rm{L}}}{V}_{\mathrm{in}}}{{C}_{{\rm{C}}}+{C}_{{\rm{P}}}},$$

where *Q*_L_ = 100 is the loaded quality factor of the resonator depicted in Fig. 1c, and r.m.s. *V*_in_ = 8.4 μV corresponding to input *P*_rf_ = −88.5 dBm. We then estimated an upper bound *α*_21_ ≥ 0.01 by rearranging equation (1), assuming *α*_R,ME_ = 0.35 ± 0.15. The resulting rf-induced fluctuation in detuning was then ${\rm{\delta }}{\varepsilon }_{{\mathrm{rms}}}^{{\mathrm{rf}}}\le 2.7\pm 0.6\,\upmu {\mathrm{eV}}$.

### Control sequence and charge noise estimation

The pulse sequence implemented to demonstrate exchange control is as follows:

1. E: (E)mpty QD Q_1_ by biasing the gate voltages such that the ground state is in the (0, 1) charge configuration, over a duration of 100 ns.
2. E–I: (I)nitialize the ground state $\left|\mathrm{S}\right\rangle$ via an adiabatic ramp from the (0, 1) to the (0, 2) configuration, over a duration of 10 μs.
3. I–S: (S)et a symmetric detuning point for applying subsequent ramps with a non-adiabatic pulse across the $\left|\mathrm{S}\right\rangle$–$\left|\mathrm{T}_{-}\right\rangle$ anticrossing to *ε* = 0, over 100 ns.
4. S–P: Initialize $\left|\downarrow \uparrow \right\rangle$ via a (P)lunge to where *J* < Δ*E*_z_ with an adiabatic ramp with respect to Δ*E*_z_ to *ε* = 0.926 meV over 250 ns.
5. P–J–P: Apply a non-adiabatic pulse to near zero detuning *ε*_J_ and back, where *J* ≫ Δ*E*_z_, for variable duration *τ*_J_.
6. P–M: Project the final state onto $\left|\mathrm{S}\right\rangle$ or $\left|\mathrm{T}_{0}\right\rangle$ for readout with a reverse adiabatic ramp to *ε* = 0.
7. M: Wait for a pre-measure delay of 6 μs, before integrating, for a measurement time of 8 μs.

The total duty cycle of the control sequence, *T*_rep_ ≈ 25 μs, provides the high-frequency bound *f*_high_ we integrate over to estimate *S*_0_, the power spectral density noise at 1 Hz. In combination with the total time used to acquire a trace of exchange oscillations, *T*_*M*_ = 1/*f*_low_ = 0.3h, we can estimate *S*_0_ from^62^

10 $${\rm{\delta }}{\varepsilon }_{\mathrm{rms}}=\sqrt{2{\int }_{{f}_{\mathrm{low}}}^{{f}_{\mathrm{high}}}{S}_{\varepsilon }(f)\,{\rm{d}}f},$$

where the power spectral density of charge noise has the functional form *S*_*ε*_(*f*) = *S*_0_/*f*^*α*^ with typical values of *α* ranging from 1 to 2. Considering the measured integrated charge noise (δ*ε*_rms_ = 5.4 μeV), we estimated upper and lower bounds of $\sqrt{{S}_{0}^{\alpha =1}}=0.91\,{\upmu}{\mathrm{eV}}/\sqrt{\,\mathrm{Hz}}$ and $\sqrt{{S}_{0}^{\alpha =2}}=0.12\,\upmu {\mathrm{eV}}/\sqrt{\,{\mathrm{Hz}}}$, respectively. This result is on par with that reported in planar MOS devices^55^ and strained Ge wells (Ge/SiGe)^63^$\sqrt{{S}_{0}}=0.6\pm 0.3\,\upmu {\mathrm{eV}}\,/\sqrt{{\mathrm{Hz}}}$, as well as Si wells in Si/SiGe heterostructures $\sqrt{{S}_{0}}=0.3\,\upmu {\mathrm{eV}}/\sqrt{{\mathrm{Hz}}}$ to $0.8\,\upmu {\mathrm{eV}}/\sqrt{{\mathrm{Hz}}}$ (refs. ^50,56^).

## Supplementary information

Supplementary Information Supplementary Sections 1–10, Figs. 1–6 and Tables 1–3.

## Data Availability

The data that support the plots within this paper and other findings of this study are available from the corresponding authors upon reasonable request.
