# Supplementary material for: Radiofrequency cascade readout of coupled spin qubits
Source: Nat Electron. 2026 Mar 30;9(3):314–23. doi: 10.1038/s41928-026-01582-8 (PMC13035474; doi:10.1038/s41928-026-01582-8)
Supplement: Supplementary file 1 — Supplementary Sections 1–10, Figs. 1–6 and Tables 1–3. [file 41928_2026_1582_MOESM1_ESM.pdf]

nature electronics

Supplementary information

https://doi.org/10.1038/s41928-026-01582-8

Radiofrequency cascade readout of coupled spin qubits

In the format provided by the authors and unedited

Contents

S1. FABRICATION OF PROCESS CONTROL MONITORS 2

S2. ELECTROSTATIC REQUIREMENTS FOR RF-DRIVEN ELECTRON CASCADE READOUT 3

S3. AMPLIFICATION FACTOR 6

S4. CHARGE STABILITY DIAGRAM SIMULATION 8

S5. MAGNETOSPECTROSCOPY SIMULATION 10

S6. RADIO-FREQUENCY ELECTRON CASCADE AS A FUNCTION OF Q_{ME} OCCUPATION 11

S7. IN-SITU DISPERSIVE READOUT PERFORMANCE 12

S8. SINGLET-TRIPLET QUBIT PERFORMANCE 12

S9. EXCHANGE ECHO PERFORMANCE 13

S10. ESTIMATED IMPROVEMENT FROM ISOTOPICALLY ENRICHED ^{28}Si 14

References 15

S1 FABRICATION OF PROCESS CONTROL MONITORS

In this Supplementary Section, we illustrate the quality of the fabrication process via electron microscope images of process control monitors (PCMs), i.e non-functional test structures integrated into the semiconductor wafer used for morphological characterisation purposes. Figure 1(a) shows a scanning electron microscopy (SEM) image of a PCM containing three gate layers and whose purpose is the creation of a linear quantum dot (QD) array. The PCM shares similarities with the array discussed in the main text. In particular, L1 gates confine the QD array to a line, L2 and L3 gates are used to control QDs, and L3 gates are used as reservoir accumulation gates. The PCM was fabricated with the same gate stack and used the same L2 gate length and pitch as the device in the main text (lateral L2 gates). The SEM image aims to illustrate features of the fabrication process such as critical dimensions, line edge roughness, and interlayer alignment accuracy.

Additionally, in Fig. 1(b), we show a cross-sectional transmission electron microscopy (TEM) image of a different PCM. The image illustrates the surface roughness of the Si-SiO_2 layer as well as the uniformity of the oxides between the gate layers. This TEM image only includes the first two gate layers.

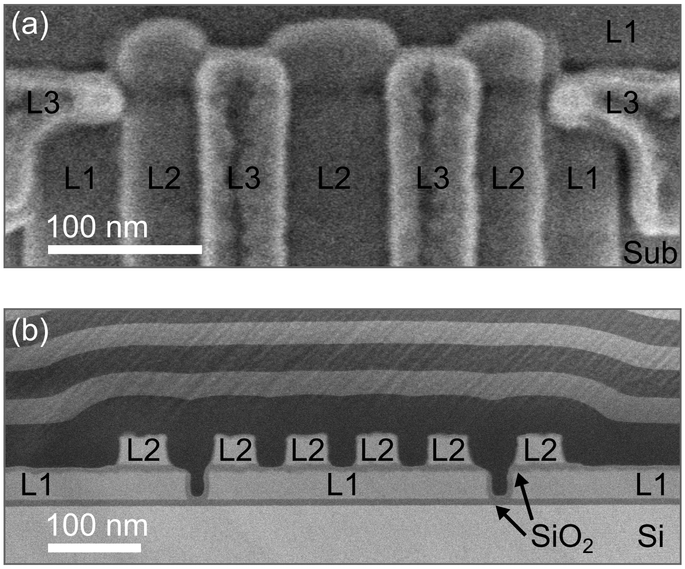

Supplementary Fig. 1. | **Fabrication process control monitors (PCMs). a** Scanning electron microscopy image of a PCM linear quantum dot array, consisting of three overlapping gate layers: L1, L2 and L3 in order from bottom to top. **b** Cross-sectional transmission electron microscopy image of a separate PCM structure to **a**, with two overlapping gate layers, buried beneath several layers of oxide. The scale bar in each panel represents 100 nm.

S2 ELECTROSTATIC REQUIREMENTS FOR RF-DRIVEN ELECTRON CASCADE READOUT

The conditions for rf-driven electron cascade are similar to those stated in a previous report on electron cascade with a proximal charge sensor [1]. It can be understood by considering the electrochemical potentials μ_i for each QD Q_i, as well as the given charge configuration $(\mathrm{N}_{\mathrm{Q}_2}, \mathrm{N}_{\mathrm{Q}_1}, \mathrm{N}_{\mathrm{Q}_{\mathrm{ME}}})$ where $\mathrm{N}_{\mathrm{Q}_i}$ refers to the number of charges in dot Q_i, as shown in Fig. 1 in the main text. For cascade to occur the DQD must be tuned to the $\mathrm{Q}_2 - \mathrm{Q}_1$ inter-dot charge transition, such that

$$\mu_{\mathrm{Q}_2}(1,1,\mathrm{N}) = \mu_{\mathrm{Q}_1}(0,2,\mathrm{N}-1). \tag{S1}$$

Note that the opposite inter-dot charge transition $\mu_{\mathrm{Q}_2}(2,0,\mathrm{N}) = \mu_{\mathrm{Q}_1}(1,1,\mathrm{N})$ also satisfies this condition. In addition to this, the reservoir-adjacent dot (Q_{ME}) must be tuned such that

$$\mu_{\mathrm{Q}_{\mathrm{ME}}}(1,1,\mathrm{N}) < 0 < \mu_{\mathrm{Q}_{\mathrm{ME}}}(0,2,\mathrm{N}), \tag{S2}$$

where the Fermi level of the reservoir is referenced to 0 and

$$\Delta\mu_{\mathrm{Q}_{\mathrm{ME}}} = \mu_{\mathrm{Q}_{\mathrm{ME}}}(0,2,\mathrm{N}) - \mu_{\mathrm{Q}_{\mathrm{ME}}}(1,1,\mathrm{N}) \gg 3.5k_{\mathrm{B}}T, \tag{S3}$$

so the shift of the Q_{ME} Coulomb oscillation due to the interdot charge transition is much larger than the Fermi broadening of the reservoir. Here k_B is the Boltzmann constant and T the temperature.

The rf mode of the casacde drive introduces an additional condition that relates to the applied rf modulation amplitude V_{rf},

$$\Delta\mu_{\mathrm{Q}_1-\mathrm{Q}_2} < eV_{\mathrm{rf}} \ll \Delta\mu_{\mathrm{Q}_{\mathrm{ME}}} \tag{S4}$$

where $\Delta\mu_{\mathrm{Q}_1-\mathrm{Q}_2} = \mu_{\mathrm{Q}_1}(1,1,\mathrm{N}) - \mu_{\mathrm{Q}_2}(0,2,\mathrm{N}-1)$. This ensures that the rf modulation only drives tunneling events between Q_2 and Q_1, without directly driving tunneling events between Q_{ME} and the reservoir.

Tuning the QD array into rf-driven electron cascade requires precise control of the electrochemical potentials of the QDs, to the order of tens of microvolts in the device presented in the main text (however, this bias range is extended to the order of a millivolt in measurements on additional devices, as noted in Supplementary Fig. 2). The tuning procedure is most clearly demonstrated in the $V_{\mathrm{G}_1} - V_{\mathrm{G}_2}$ gate-voltage-space, as shown in Extended Data Fig. 1 and Supplementary Fig. 2, where in each panel $V_{\mathrm{G}_{\mathrm{S}}}$ is varied, bringing the system into and out of cascade. In this configuration, the range of $V_{\mathrm{G}_{\mathrm{S}}}$ bias voltages satisfying the conditions for cascade are given by $V_{\mathrm{G}_{\mathrm{S}}}^{casc} = 728.6 \pm 0.1$ mV (Extended Data Fig. 1), a relatively narrow range of voltages as compared to the 14 mV addition voltage of Q_{ME}.

Extended Data Figure 1 (a) and (e) shows two limiting cases, in which the $\mu_{\mathrm{Q}_{\mathrm{ME}}}(0,2,\mathrm{N})$ or $\mu_{\mathrm{Q}_{\mathrm{ME}}}(1,1,\mathrm{N})$ potentials do not meet the condition set by Eq. (S2), i.e. the levels are below or above the Fermi level of the reservoir, respectively. Similar cases are observed in panels (b) and (d), where $\mu_{\mathrm{Q}_{\mathrm{ME}}}(0,2,\mathrm{N})$ or $\mu_{\mathrm{Q}_{\mathrm{ME}}}(1,1,\mathrm{N})$ now just align with the Fermi level in the reservoir but are within its Fermi broadening. In panel (b), the Q_{ME} transition is present in the (0, 2, N-1) − (0, 2, N) occupation regime, but absent in the (1, 1, N-1) − (1, 1, N) regime. Likewise, in panel (d), the Q_{ME} transition is present in the (1, 1, N-1) − (1, 1, N) regime but absent in the (0, 2, N-1) − (0, 2, N) regime. The contrast in signal between the different charge occupations shown in Extended Data Fig. 1(b) and (d) is well-suited to standard charge sensing. It is only in panel (c), when the sequential tunneling event occurs, the cascade conditions are met and we observe the enhanced intensity of the interdot charge transition.

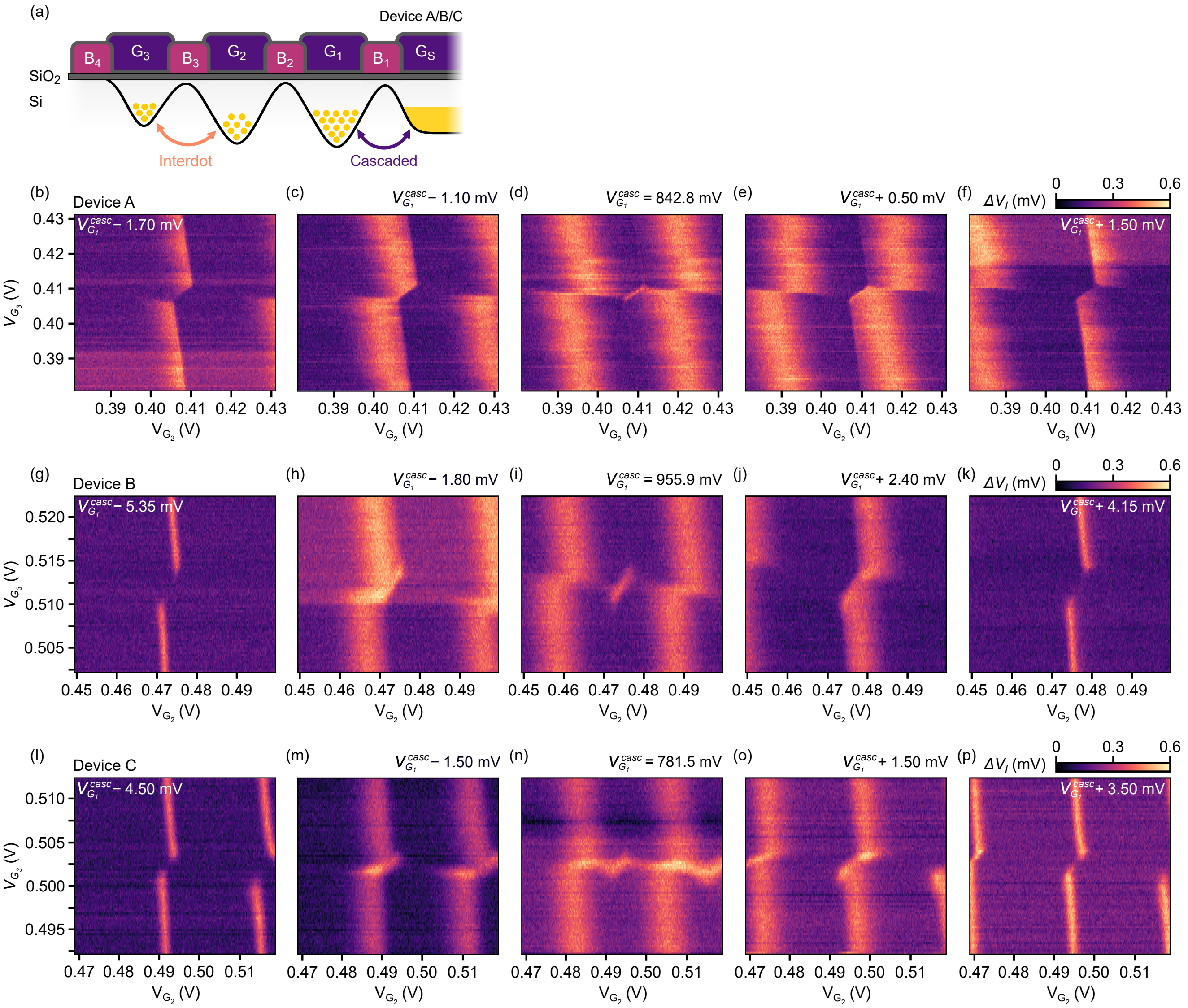

Supplementary Fig. 2. | **Radio-frequency cascade reproduced across multiple devices. a** Cross-section schematic of the three quantum dot arrays, A, B, and C, where each array has varied gate dimensions. The exact number of electrons occupying each QD is unknown. Each row depicts each of the devices being tuned around the cascade regime (centre column) for: **b-f** Device A ($V_{\mathrm{GS}}^{\mathrm{casc}} = 842.8 \pm 1.0$ mV), **h-l** Device B ($V_{\mathrm{GS}}^{\mathrm{casc}} = 955.9 \pm 1.2$ mV), **m-p** Device C ($V_{\mathrm{GS}}^{\mathrm{casc}} = 781.5 \pm 1.5$ mV).

In panels (f-j), we present matching radio-frequency simulations of the triple QD system that highlight the enhanced intensity of the interdot charge transition (see Section. S4). Further, we supplement the explanation with schematics of the electrochemical levels in each V_{GS} conditions in panels (k-o).

Supplementary Figure 2, shows the rf-cascade tuning process across three additional devices (A,B, and C), where each device is a linear array of QDs similar to the device discussed in the main text, but with the addition of inter-dot barrier gates. The barriers enable finer control of inter-dot coupling and broaden the cascade window from tens of microvolts to the order of a millivolt. Each

row illustrates the same tuning sequence as Extended Data Fig. 1, as V_{G_S} is increased $\mu_{\mathrm{Q_{ME}}}$ is tuned with respect to the Fermi level of the electron reservoir, in and out of the cascade regime. The cascade window for each device is defined by $V_{\mathrm{G_S}}^{\mathrm{A,casc}} = 842.8 \pm 1.0$ mV, $V_{\mathrm{G_S}}^{\mathrm{B,casc}} = 955.9 \pm 1.2$ mV, and $V_{\mathrm{G_S}}^{\mathrm{C,casc}} = 781.5 \pm 1.5$ mV respectively.

S3 AMPLIFICATION FACTOR

Here, we obtain the expression for the signal amplification factor generated by the cascade process, i.e. Eq. (1) in the main text. To determine the amplification factor, we consider two different charge movement events as seen from the electrode connected to the resonator, in this case, the electron reservoir (R):

1. **In-situ dispersive readout** involving solely a charge transition between Q_1 and Q_2.
2. **Cascade readout** involving (1) plus the cascaded charge transition between Q_{ME} and the reservoir.

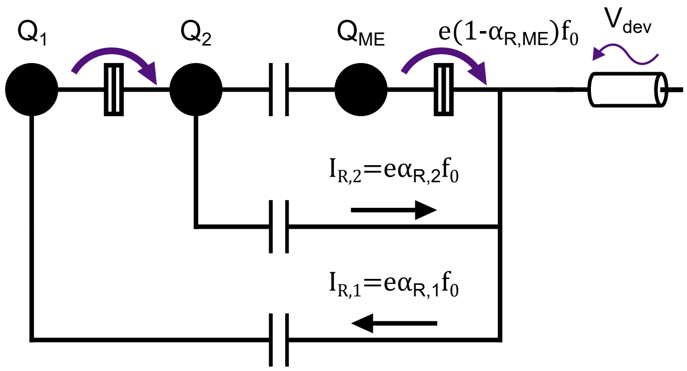

Supplementary Fig. 3. | **Schematic circuit depicting alternating current induced by the radio-frequency driven electron cascade.** Two tunneling processes are depicted between tunnel coupled (1) quantum dots Q_1 and Q_2 and (2) Q_{ME} and the reservoir. The induced alternating current at the reservoir from each of these tunneling events is given by $I_{\mathrm{R,i}} = e\alpha_{R,i} f_0$ where $i = 1, 2, \mathrm{ME}$ and $\alpha_{\mathrm{R,i}}$ are the corresponding lever arms, with rf drive frequency f_0. The rf modulation applied to the system is represented by V_{dev}, the device voltage.

To obtain the amplification factor, we first consider the expressions for the signal-to-noise ratio for case (1) [2]. In particular, we assume that the system is in the low frequency limit $hf_{\mathrm{rf}} \ll \Delta_{\mathrm{c}}$ (where f_{rf} is the frequency of the resonator and $\Delta_{\mathrm{c}} = 2t_c$), the small signal regime $Q_{\mathrm{L}}\Delta C_{\mathrm{Q}}/(2C_{\mathrm{tot}}) \ll 1$ (where Q_{L} is the loaded quality factor of the resonator, ΔC_{Q} is the change in quantum capacitance of the system and C_{tot} the total capacitance of the system) and the large excitation regime, $\alpha_{21} e V_{\mathrm{dev}} \gg \Delta_{\mathrm{c}}$ to ensure an electron tunnels every half cycle of the rf excitation. Here $\alpha_{21} = \alpha_{\mathrm{R,2}} - \alpha_{\mathrm{R,1}}$ is the interdot lever arm as seen from the reservoir and V_{dev} is the amplitude of the oscillatory voltage arriving at the reservoir. In this case, the SNR is

$$\mathrm{SNR}_{21} \propto \frac{(\alpha_{21} e)^2}{k_{\mathrm{B}} T_{\mathrm{n}}} Q_0 Z_{\mathrm{r}} f_{\mathrm{rf}}^2 = \frac{I_{\mathrm{R,21}}^2 R}{k_{\mathrm{B}} T_{\mathrm{n}}}, \tag{S5}$$

where T_{n} is the noise temperature of the system, $Q_0 = R\sqrt{C_{\mathrm{p}}/L}$ is the internal quality factor of the resonator, $Z_{\mathrm{r}} = \sqrt{L/C_{\mathrm{p}}}$ is the resonator impedance, R is the resistance (in parallel with L and C_{p}) representing the losses in the resonator, and $I_{\mathrm{R,21}}$ is the AC current amplitude produced at the reservoir by the oscillatory charge motion between Q_1 and Q_2. Note that $\alpha_{21} e f_{\mathrm{rf}} = I_{\mathrm{R,21}}$ is the induced current at the reservoir due to the cyclic interdot tunneling.

We now perform the same calculation for the charge transition between the multi-electron QD Q_{ME} and the reservoir R, a dot-to-reservoir transition [3]. Again, considering the small signal regime, the

large excitation regime, $\alpha_{\mathrm{R,ME}} e V_{\mathrm{dev}} \gg k_{\mathrm{B}} T$ (where $\alpha_{\mathrm{R,ME}}$ is the lever arm from the reservoir to $\mathrm{Q_{ME}}$ and T is the electron temperature) and considering additionally the fast tunneling regime $\gamma \gg f_{\mathrm{rf}}$ (where γ is the tunnel rate between $\mathrm{Q_{ME}}$ and R), we obtain

$$\mathrm{SNR_{ME}} \propto \frac{(1-\alpha_{\mathrm{R,ME}})^2 e^2}{k_{\mathrm{B}} T_{\mathrm{n}}} Q_0 Z_{\mathrm{r}} f_{\mathrm{rf}}^2 = \frac{I_{\mathrm{R,ME}}^2 R}{k_{\mathrm{B}} T_{\mathrm{n}}}. \tag{S6}$$

Here, the resonator is coupled to Q_{ME} via a tunnel barrier, rather than a capacitively coupled gate, and hence the induced charge is $(1-\alpha_{\mathrm{R,ME}})e$.

After this analysis, we highlight a critical result: the SNR is proportional to the square of the AC current amplitude produced by the relevant process, whereas the other two parameters, R and T_{n}, are independent of the charge transfer process, particularly in the typical regime where the noise temperature is determined by the first amplifying stage.

From this we can determine an expression for the amplification factor by considering the two tunneling processes presented in Supplementary Fig. 3. In process (1) the alternating current generated by cyclical tunneling from $\mathrm{Q_1}$ to $\mathrm{Q_2}$ is,

$$I_{\mathrm{R,21}} = \alpha_{21} e f_{\mathrm{rf}} = (\alpha_{\mathrm{R,2}} - \alpha_{\mathrm{R,1}}) e f_{\mathrm{rf}}. \tag{S7}$$

In process (2), in addition to the current produced by process (1), we have the current produced by the cascade process adding to a total of,

$$I_{\mathrm{cascade}} \approx I_{\mathrm{R,21}} + I_{\mathrm{R,ME}} = (\alpha_{\mathrm{R,2}} - \alpha_{\mathrm{R,1}}) e f_{\mathrm{rf}} + (1-\alpha_{\mathrm{R,ME}}) e f_{\mathrm{rf}}. \tag{S8}$$

The amplification factor A is then given by the ratio between the two processes,

$$A = \frac{I_{\mathrm{cascade}}}{I_{\mathrm{R,21}}} = 1 + \frac{1-\alpha_{\mathrm{R,ME}}}{\alpha_{\mathrm{R,2}} - \alpha_{\mathrm{R,1}}}. \tag{S9}$$

S4 CHARGE STABILITY DIAGRAM SIMULATION

To simulate the cascade phenomena we observe in the main text, we calculate the charge stability diagram for a specific voltage configuration using the Constant Interaction Model [4]. The energies in this model are defined as

$$E = \frac{1}{2}\vec{V}^T \mathbf{C}_{\mathbf{cc}}^{-1}\vec{V} \tag{S10}$$

where $\mathbf{C}_{\mathbf{cc}}$ is the capacitance matrix for the QDs, containing the mutual capacitance between each pair

$$C_{cc} = \begin{pmatrix} 35.000 & -4.882 & -1.886 \\ -4.882 & 27.936 & -0.402 \\ -1.886 & -0.402 & 42.048 \end{pmatrix} \tag{S11}$$

where each element is given in aF and $\vec{V} = e(\mathbf{C}_{\mathbf{cv}}\vec{V_G} - |e|\vec{N})$. Additionally we define $\mathbf{C}_{\mathbf{cv}}$ to represent the capacitance matrix governing the interactions between the gate and charges, containing the capacitance between each QD and the corresponding gates,

$$C_{cv} = \begin{pmatrix} 0.898 & 0.146 & 0.000 \\ 0.537 & 0.453 & 0.000 \\ 0.063 & 0.020 & 2.016 \end{pmatrix} \tag{S12}$$

where each element is given in aF. The number of charges on each dot are given by $\vec{N}$, and $\vec{V_G}$ denotes the applied gate voltages. Throughout the simulations, we use natural units and set the charge of the electron $e = 1$. We note that the ratio between capacitances in the matrices defined here are found to be in qualitative agreement with the data in Extended Data Fig. 1(a-e), however the magnitude of these capacitances have not been verified experimentally.

Without loss of generality, we assume that an M-quantum dot array can exist in a state from a set of L Fock states, denoted as $\mathcal{F} = \{\Lambda_j = (\lambda_{1,j}, \ldots, \lambda_{M,j}) \mid \lambda_{i,j} \in \mathbb{Z}^+, \forall j = 1, 2, \ldots, L\}$. Here $\lambda_{i,j}$ represents the occupancy number of QD i in Fock state Λ_j.

In radio-frequency reflectometry, the measured signal is directly proportional to the change in capacitance of the system, this can be described mathematically as

$$\Delta C_j = \frac{dQ_T}{dV_j}, \tag{S13}$$

here Q_T denotes the total charge of the system and V_j represents the jth gate. We take inspiration from [5] and rewrite the change in capacitance as measured from gate V_j to be

$$\Delta C_j = \sum_{i=1}^{M} C_{i,j} = e\sum_{i=1}^{M} \alpha_{i,j}\frac{d\langle n_i\rangle}{dV_j}. \tag{S14}$$

In this context, $C_{i,j}$ represents the capacitance felt from dot i by gate j. The average occupancy of dot i is denoted $\langle n_i\rangle$. The lever arm matrix is defined as $\alpha = \mathbf{C}_{\mathbf{cc}}^{-1}\mathbf{C}_{\mathbf{cv}}$, which links gate-induced potential changes to the charge states of the QDs. To calculate $\langle n_i\rangle$, we iterate through each Fock state in $\mathcal{F}$ and compute its corresponding probability. Subsequently, a weighted sum of the occupation numbers of each Fock state at position i is performed, expressed as

$$\langle n_i\rangle = \sum_{k=1}^{L} \lambda_{i,k} \cdot P_k, \tag{S15}$$

where P_k represents the probability of the QD array being in the Fock state Λ_k. We assume a Boltzmann distribution and write the probability accordingly;

$$P_k = \frac{1}{Z}\exp\left(-\frac{\epsilon_k}{k_b T}\right) = \frac{\exp\left(-\frac{\epsilon_k}{k_b T}\right)}{\sum_{l=1}^{L}\exp\left(-\frac{\epsilon_l}{k_b T}\right)} \tag{S16}$$

where Z represents the partition function, and ϵ_k represents the energy required for QD array to be in the Fock state Λ_k. Substituting this into equation S15, we obtain;

$$\langle n_i \rangle = \sum_{k=1}^{L} \lambda_{i,k} \cdot \frac{\exp\left(-\frac{\epsilon_k}{k_b T}\right)}{\sum_{l=1}^{L}\exp\left(-\frac{\epsilon_l}{k_b T}\right)} \tag{S17}$$

This term can be further substituted into equation S14, to obtain;

$$\Delta C_{j,\mathrm{tot}} = \sum_{i=1}^{M} C_{i,j} \tag{S18}$$

$$= e\sum_{i} \alpha_{i,j} \frac{d}{dV_j}\left(\sum_{k=1}^{L} \lambda_{i,k} \cdot \frac{\exp\left(-\frac{\epsilon_k}{k_b T}\right)}{\sum_{l=1}^{L}\exp\left(-\frac{\epsilon_l}{k_b T}\right)}\right) \tag{S19}$$

We use the above formalism to simulate the charge stability diagrams in the $V_{\mathrm{G}_1} - V_{\mathrm{G}_2}$ gate-voltage space. This can be seen in Extended Data Fig. 1(f-j), where different voltage configurations were applied to the multi-electron dot via $V_{\mathrm{G}_\mathrm{S}}$.

S5 MAGNETOSPECTROSCOPY SIMULATION

Here, we describe the simulations of the magnetospectroscopy map in the inset of Fig. 1(c) in the main text. that allow us to estimate both the tunnel coupling t_{c} and the electron temperature T_{e}. We utilize the simplified Hamiltonian:

$$H = \frac{1}{2}\begin{pmatrix} \varepsilon & \Delta_{\mathrm{c}} & 0 & 0 & 0 \\ \Delta_{\mathrm{c}} & -\varepsilon & 0 & 0 & 0 \\ 0 & 0 & -\varepsilon - \hat{B} & 0 & 0 \\ 0 & 0 & 0 & -\varepsilon & 0 \\ 0 & 0 & 0 & 0 & -\varepsilon + \hat{B} \end{pmatrix}, \tag{S20}$$

where $\hat{B} = 2g\mu_{\mathrm{B}}B$, g is the electron g-factor (which we approximate to 2 for both QDs). Then, we calculate the quantum capacitance of the system, C_{Q}, given by,

$$C_{\mathrm{Q}} = -\sum_i (e\alpha)^2 \frac{\partial^2 E_i}{\partial \varepsilon^2} P_i^{\mathrm{th}}. \tag{S21}$$

where E_i are the eigenenergies of the above Hamiltonian and P_i^{th} is the thermal probability of the state i,

$$P_i^{\mathrm{th}} = \exp(-E_i/k_{\mathrm{B}}T_{\mathrm{e}})/Z. \tag{S22}$$

Here, T_{e} is the DQD temperature and Z is the partition function over all states [6]. We plot the results of the simulations in Supplementary Fig. 4 where, in panel (a) and (b), we show the energy spectrum of the system as a function of detuning for $B = 0.5$ T and 0 T, respectively, and in panel (c), we plot the normalised quantum capacitance of the systems as a function of ε and B. We find that the best match between the data and simulations occurs when $t_{\mathrm{c}} = 2.4$ GHz and $T_{\mathrm{e}} = 50$ mK.

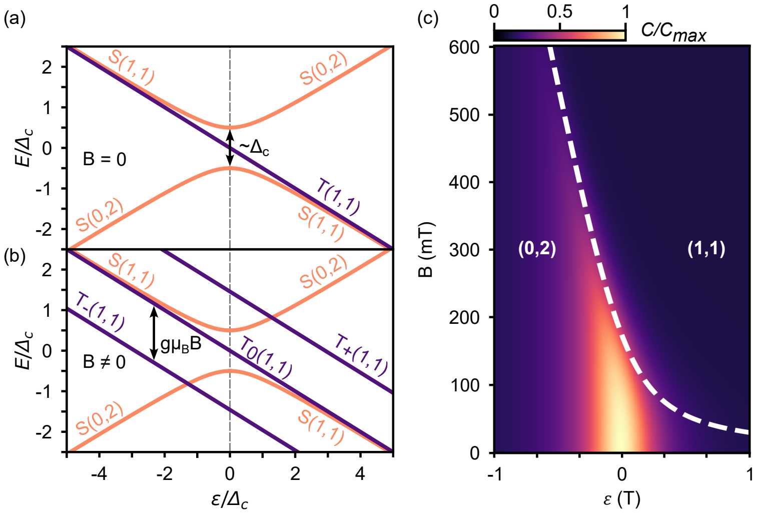

Supplementary Fig. 4. | **Magneto-spectroscopy simulation of the (0,2)-(1,1) inter-dot charge transition**. Eigenenergies of the two-electron spin states, singlet $|\mathrm{S}\rangle$ and triplet $|\mathrm{T}\rangle$ states as a function of detuning ε normalised by tunnel coupling $\Delta = 2t_c$. For **a**, applied magnetic field $B = 0$ mT and **b** $B = 500$ mT. **c** Normalised quantum capacitance simulation versus detuning and magnetic field, with the degeneracy point of the $|\mathrm{S}\rangle$-$|\mathrm{T}_-\rangle$ crossing overlaid (white dashed line).

S6 RADIO-FREQUENCY ELECTRON CASCADE AS A FUNCTION OF Q_{ME} OCCUPATION

We observe that the occupancy of Q_{ME}, does not have a significant effect on the cascade signal. Supplementary Figure 5 shows the charge stability diagrams measured at different occupations N_{ME}, in each case we see the cascaded interdot charge transition (Fig S5(a-b)). Additionally, we observe Pauli spin blockade in each configuration, as evident from the magnetospectroscopy measurements in Fig. S5(c-d), where the disappearance of signal for increasing $|B|$ indicates the $|S\rangle$ is no longer in the ground state at $\varepsilon = 0$.

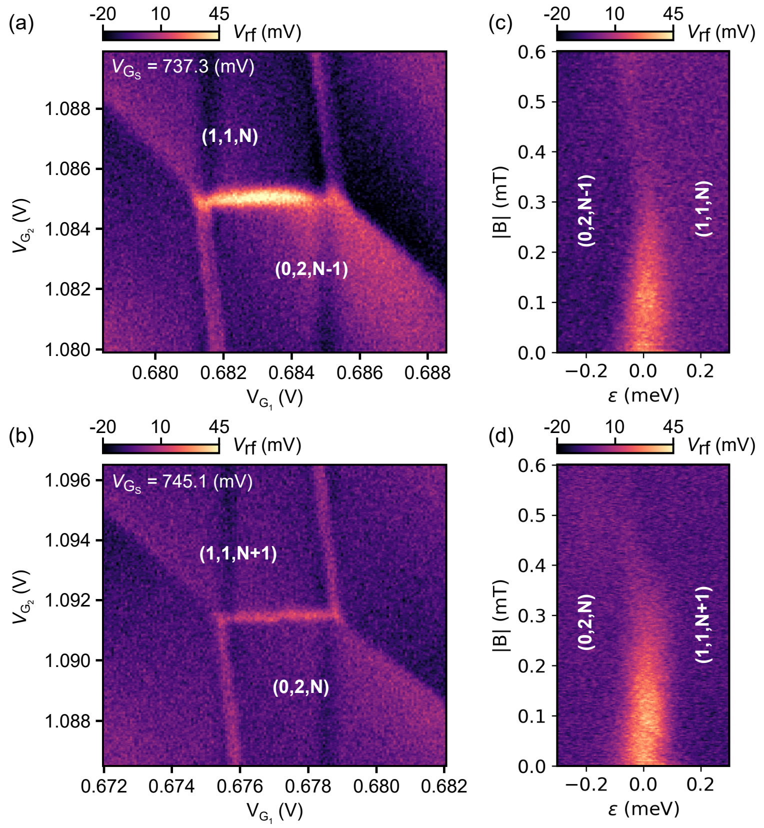

Supplementary Fig. 5. | **Radio-frequency driven electron cascade at different Q_{ME} occupations.** Charge stability diagrams **(a-b)** and magnetospectroscopy **(c-d)** for Q_{ME} transitions, $(N_{ME} - 1) \rightarrow (N_{ME})$ and $(N_{ME}) \rightarrow (N_{ME} + 1)$, respectively. *We note that the cascade observed in the charge stability diagram in panel (**b**) is not optimally tuned, but it is in the corresponding magnetospectroscopy measurement in panel **(d)**. Furthermore, the dark lines observed in the charge stability diagrams, at $V_{G_2} = 0.682$ V and 0.685 V in panel **(a)**,are the result of the AC-coupled measurement mode.

S7 IN-SITU DISPERSIVE READOUT PERFORMANCE

Reference	Platform	Resonator	τ_{int} (µs)	$\mathcal{F}_r$ (%)	T_1 (µs)
Pakkiam *et al.* 2018 [7]	Donor in Si	Off-chip SC	300	82.9	620
West *et al.* 2019 [8]	Planar Si/SiO_2	SMD	2000	73.3	4500
Zheng *et al.* 2019 [9]	Si/SiGe	On-chip SC	6	98.0	159
Chittock-Wood *et al.* 2025	Planar Si/SiO_2	Off-chip SC	8	67.0	24

Supplementary Table I. **In-situ dispersive readout performance across different silicon technology platforms.** Here resonators are categorised into three-types, on- and off-chip superconducting (SC) resonators and those constructed using surface mounted (SMD) circuit components.

S8 SINGLET-TRIPLET QUBIT PERFORMANCE

Reference	Platform	Q	τ_{gate} (ns)
Dial *et al.* 2013 [10]	GaAs/AlGaAs	6	0.6
Wu *et al.* 2014 [11]	Si/SiGe	1.5	36
Higginbotham *et al.* 2014 [12]	GaAs/AlGaAs	15	0.3
Jang *et al.* 2020 [13]	GaAs/AlGaAs	26	1.1
Jirovec *et al.* 2021 [14]	Ge/SiGe	50	8
Jock *et al.* 2018 [15]	$^{28}Si/SiO_2$	2	250
Connors *et al.* 2022 [16]	Si/SiGe	5.3	17
Chittock-Wood *et al.* 2025	Si/SiO_2	$\geq$10	22

Supplementary Table II. **Singlet-triplet qubit quality factors and gate times across different semiconductor platforms,** where $Q = T_2^*\Omega$ is the qubit quality factor calculated using the qubit frequency Ω and dephasing time T_2^*, and $\tau_{\text{gate}} = 1/2\Omega$ is the qubit gate time.

S9 EXCHANGE ECHO PERFORMANCE

Reference	Platform	$\delta\varepsilon_{\rm rms}$ (μeV)	$\Delta E_{\rm z,rms}$ (neV)	T_2^* (μs)	$T_2^{\rm echo}$ (μs)	$T_{2,\rm mag}^*$ (μs)
Jock *et al.* 2018 [15]	$^{28}Si/SiO_2$	2.0	$\leq 0.2^a$	1.00	8.4	≥20
Connors *et al.* 2022 [16]	Si/SiGe	2.7	$\leq 1.3^b$	0.10	0.4	≥9
Chittock-Wood *et al.* 2025	Si/SiO_2	5.4	3.4	0.04	0.4	3

Supplementary Table III. **Exchange echo reports in silicon quantum devices,** where a singlet-triplet qubit is configured to dephase under exchange J and is refocused via the difference in Zeeman energy between the two dots ΔE_z [10] (in a DQD). Noise parameters $\delta\varepsilon_{\rm rms}$ and $\delta\Delta E_{\rm z,rms}$ describe rms fluctuations in detuning ε and ΔE_z, respectively. Note that here, the dephasing time T_2^* listed does not represent the longest T_2^* achieved in each reference, but is the reported value at the detuning $\varepsilon_{\rm echo}$ where the echo was implemented. The echo time is reported as $T_2^{\rm echo}$, and this is contrasted against the magnetic limited dephasing time $T_{2,\rm mag}^* = \lim_{\delta\varepsilon_{\rm rms}\to 0} T_2^*$, $^{a-b}$determined by $\sqrt{2}\hbar/\lim_{\varepsilon\to\infty} T_2^*$ in ^{a}Fig. 4(d) [15] and ^{b}Fig. 2(d) [16].

S10 ESTIMATED IMPROVEMENT FROM ISOTOPICALLY ENRICHED ^{28}Si

The random Overhauser fluctuations in this effective magnetic field are then described by,

$$\delta\mathcal{A} = \frac{\mathcal{A}}{\sqrt{N_{\text{nuc}}}} = \frac{\hbar}{T^*_{2,m}}, \tag{S23}$$

it then follows that the rms magnetic noise calculated in the main text is equivalent to $\delta\Delta E_{z,rms} = \sqrt{2}\delta\mathcal{A}$, we therefore find $\delta\mathcal{A} = 2.4 \pm 0.3$ neV (21 ± 3 μT). The corresponding number of ^{29}Si nuclei that an electron interacts can then be obtained by rearranging eq. (S23) such that,

$$N_{\text{nuc}} = \left(\frac{\hbar}{\mathcal{A}_0 T^*_{2,m}}\right)^2 \tag{S24}$$

where $\mathcal{A}_0 = \mathcal{A}/N_{\text{nuc}} = 0.043$ neV is the HFI energy per nuclei for Si [17]. We find $N_{\text{nuc}} = 3100 \pm 800$ for the $T^*_{2,m}$ measured in the main text, which corresponds to the 4.67% natural abundance of ^{29}Si [18]. Furthermore, we can estimate the volume of the QD from,

$$V_{\text{QD}} = \frac{N_{\text{nuc}}}{f_{^{29}\text{Si}}\rho_{\text{atom}}}, \tag{S25}$$

where $\rho_{\text{atom}} = 4.99 \times 10^{28}$ m^{-3} is the atomic density of Si, and $f_{^{29}\text{Si}}$ is the fraction of ^{29}Si nuclei per m^{-3}, indicating a QD volume of $V_{\text{QD}} = 1300 \pm 300$ nm^3. Then to calculate $T^*_{2,m}$ for 800 ppm ^{29}Si, we substitute $f_{^{29}\text{Si}} = 0.08\% \times 0.0467\%$ into eq. (S25) and re-arrange to find $N^{800\text{ppm}}_{\text{nuc}} = 2 \pm 1$. Finally, substituting this result in eq. (S23) we find $T^*_{2,m} = \hbar/\mathcal{A}_0\sqrt{N^{800\text{ppm}}_{\text{nuc}}} = 9.8 \pm 0.8$ μs, corresponding to an improvement of an order of magnitude. This is consistent with studies of similar two-electron systems defined in 800 ppm isotopically purified planar ^{28}Si/SiO$_2$ devices, where $T^*_2 \approx 1 - 3$ μs [15, 19–21]

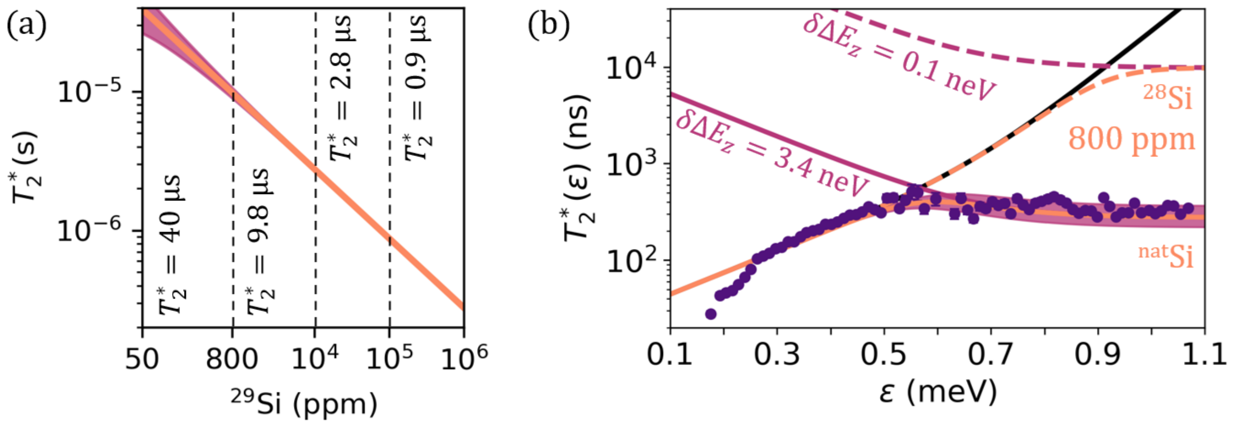

Supplementary Fig. 6. | **Expected extension to T^*_2 from isotopic purification. a**, Extrapolated dephasing time T^*_2 as a function of ^{29}Si isotopic abundance in the silicon substrate, in units of parts per million (ppm). **b**, Dephasing time as a function of detuning ε, with dataset for a natural silicon substrate and fits using Eq. (4) (solid orange line), with magnetic noise and electron noise contributions to $T^*_2(\varepsilon)$ depicted by solid light purple and black lines respectively. The dashed lines show the corresponding estimate for a 800 ppm isotopically enriched silicon substrate. Shaded area shows the propagated error from the ±1 standard deviation of fit parameters.

[1] C. J. van Diepen, T.-K. Hsiao, U. Mukhopadhyay, C. Reichl, W. Wegscheider, and L. M. K. Vandersypen, Electron cascade for distant spin readout, *Nat. Commun* **12**, 77 (2021).

[2] F. Vigneau, F. Fedele, A. Chatterjee, D. Reilly, F. Kuemmeth, M. F. Gonzalez-Zalba, E. Laird, and N. Ares, Probing quantum devices with radio-frequency reflectometry, *Applied Physics Reviews* **10**, 021305 (2023).

[3] G. A. Oakes, V. N. Ciriano-Tejel, D. F. Wise, M. A. Fogarty, T. Lundberg, C. Lainé, S. Schaal, F. Martins, D. J. Ibberson, L. Hutin, B. Bertrand, N. Stelmashenko, J. W. A. Robinson, L. Ibberson, A. Hashim, I. Siddiqi, A. Lee, M. Vinet, C. G. Smith, J. J. L. Morton, and M. F. Gonzalez-Zalba, Fast high-fidelity single-shot readout of spins in silicon using a single-electron box, *Phys. Rev. X* **13**, 011023 (2023).

[4] W. G. van der Wiel, S. D. Franceschi, J. M. Elzerman, T. Fujisawa, S. Tarucha, and L. P. Kouwenhoven, Electron transport through double quantum dots, Rev. Mod. Phys. **75**, 1 (2002).

[5] R. Mizuta, R. M. Otxoa, A. C. Betz, and M. F. Gonzalez-Zalba, Quantum and tunneling capacitance in charge and spin qubits, Phys. Rev. B **95**, 045414 (2017).

[6] T. Lundberg, J. Li, L. Hutin, B. Bertrand, D. J. Ibberson, C.-M. Lee, D. J. Niegemann, M. Urdampilleta, N. Stelmashenko, T. Meunier, J. W. A. Robinson, L. Ibberson, M. Vinet, Y.-M. Niquet, and M. F. Gonzalez-Zalba, Spin quintet in a silicon double quantum dot: Spin blockade and relaxation, Phys. Rev. X **10**, 041010 (2020).

[7] P. Pakkiam, A. V. Timofeev, M. G. House, M. R. Hogg, T. Kobayashi, M. Koch, S. Rogge, and M. Y. Simmons, Single-shot single-gate rf spin readout in silicon, Phys. Rev. X **8**, 041032 (2018).

[8] A. West, B. Hensen, A. Jouan, T. Tanttu, C.-H. Yang, A. Rossi, M. F. Gonzalez-Zalba, F. Hudson, A. Morello, D. J. Reilly, and A. S. Dzurak, Gate-based single-shot readout of spins in silicon, Nat. Nanotechnol. **14**, 437 (2019).

[9] G. Zheng, N. Samkharadze, M. L. Noordam, N. Kalhor, D. Brousse, A. Sammak, G. Scappucci, and L. M. K. Vandersypen, Rapid gate-based spin read-out in silicon using an on-chip resonator, Nat. Nanotechnol. **14**, 742 (2019).

[10] O. E. Dial, M. D. Shulman, S. P. Harvey, H. Bluhm, V. Umansky, and A. Yacoby, Charge noise spectroscopy using coherent exchange oscillations in a singlet-triplet qubit, Phys. Rev. Lett. **110**, 146804 (2013).

[11] X. Wu, D. R. Ward, J. R. Prance, D. Kim, J. K. Gamble, R. T. Mohr, Z. Shi, D. E. Savage, M. G. Lagally, M. Friesen, S. N. Coppersmith, and M. A. Eriksson, Two-axis control of a singlet–triplet qubit with an integrated micromagnet, Proc. Natl. Acad. Sci. U.S.A. **111**, 11938 (2014).

[12] A. P. Higginbotham, F. Kuemmeth, M. P. Hanson, A. C. Gossard, and C. M. Marcus, Coherent operations and screening in multielectron spin qubits, Phys. Rev. Lett. **112**, 026801 (2014).

[13] W. Jang, J. Kim, M.-K. Cho, H. Chung, S. Park, J. Eom, V. Umansky, Y. Chung, and D. Kim, Robust energy-selective tunneling readout of singlet-triplet qubits under large magnetic field gradient, npj Quantum Information **6**, 64 (2020).

[14] D. Jirovec, A. Hofmann, A. Ballabio, P. M. Mutter, G. Tavani, M. Botifoll, A. Crippa, J. Kukucka, O. Sagi, F. Martins, J. Saez-Mollejo, I. Prieto, M. Borovkov, J. Arbiol, D. Chrastina, G. Isella, and G. Katsaros, A singlet-triplet hole spin qubit in planar Ge, *Nat. Mater.* **20**, 1106 (2021).

[15] R. M. Jock, N. T. Jacobson, P. Harvey-Collard, A. M. Mounce, V. Srinivasa, D. R. Ward, J. Anderson, R. Manginell, J. R. Wendt, M. Rudolph, T. Pluym, J. K. Gamble, A. D. Baczewski, W. M. Witzel, and M. S. Carroll, A silicon metal-oxide-semiconductor electron spin-orbit qubit, *Nat. Commun* **9**, 1768 (2018).

[16] E. J. Connors, J. Nelson, L. F. Edge, and J. M. Nichol, Charge-noise spectroscopy of si/sige quantum dots via dynamically-decoupled exchange oscillations, Nature Communications **13**, 940 (2022).

[17] L. V. C. Assali, H. M. Petrilli, R. B. Capaz, B. Koiller, X. Hu, and S. Das Sarma, Hyperfine interactions in silicon quantum dots, Phys. Rev. B **83**, 165301 (2011).

[18] G. Audi, O. Bersillon, J. Blachot, and A. Wapstra, The nubase evaluation of nuclear and decay properties, Nuclear Physics A **624**, 1 (1997).

[19] P. Harvey-Collard, N. T. Jacobson, M. Rudolph, J. Dominguez, G. A. Ten Eyck, J. R. Wendt, T. Pluym, J. K. Gamble, M. P. Lilly, M. Pioro-Ladrière, and M. S. Carroll, Coherent coupling between a quantum dot and a donor in silicon, Nature Communications **8**, 1029 (2017).

[20] M. A. Fogarty, K. W. Chan, B. Hensen, W. Huang, T. Tanttu, C. H. Yang, A. Laucht, M. Veldhorst, F. E. Hudson, K. M. Itoh, D. Culcer, T. D. Ladd, A. Morello, and A. S. Dzurak, Integrated silicon qubit platform with single-spin addressability, exchange control and single-shot singlet-triplet readout, Nat. Commun **9**, 4370 (2018).

[21] R. M. Jock, N. T. Jacobson, M. Rudolph, D. R. Ward, M. S. Carroll, and D. R. Luhman, A silicon singlet–triplet qubit driven by spin-valley coupling, Nat. Commun **13**, 641 (2022).
